# Childhood family socioeconomic status is linked to adult brain electrophysiology

**DOI:** 10.1371/journal.pone.0307406

**Published:** 2024-08-20

**Authors:** Elif Isbell, Nancy E. Rodas De León, Dylan M. Richardson

**Affiliations:** Department of Psychological Sciences, University of California Merced, Merced, California, United States of America; Universität Hamburg, GERMANY

## Abstract

A large body of research has linked childhood family socioeconomic status (SES) to neurodevelopment in childhood and adolescence. However, it remains unclear to what extent childhood family SES relates to brain functioning in adulthood. To address this gap, the present study investigated the associations between retrospective accounts of objective and subjective childhood family SES and two well-established electrophysiological indices of brain functioning in adulthood—the MMN and P3b event-related potentials (ERP) components, as neural correlates of automatic change detection and cognitive control respectively. Higher objective childhood family SES, as proxied by parent educational attainment in childhood, was associated with larger (more positive) P3b amplitudes in adulthood. In contrast, there was no association between childhood parent educational attainment and the magnitude of MMN. Adult reports of subjective family SES during childhood were not related to the magnitude of MMN or P3b. These findings suggest that the links between childhood parent educational attainment and brain functioning may extend into adulthood, especially for brain functions supporting cognitive control. These results also imply that, when using retrospective accounts of childhood family SES, objective and subjective reports likely proxy different childhood experiences that have distinct links with specific neurodevelopmental outcomes, and that some of these links may not persist into adulthood. Our findings lay the groundwork for future investigations on how and why childhood family SES relates to brain functioning in adulthood.

## Introduction

A growing body of research has linked childhood family socioeconomic status (SES) to neurodevelopment in childhood and adolescence [1–3]. However, we know very little about the extent to which childhood family SES is linked to brain functioning in adulthood. Addressing this gap is crucial for advancing our knowledge of how brain functions develop in adaptation to life experiences and what factors constitute risk and protection for brain functioning across the lifespan. To contribute to closing this gap, the main aim of our study was to investigate the links between retrospective accounts of objective and subjective childhood family SES and brain functions supporting perception and cognition in young adults.

### Context of childhood family SES

Childhood family SES is typically assessed through objective indicators such as parent educational attainment, occupational prestige, and household income, as well as subjective evaluations of social status by parents or children and adolescents themselves [4, 5]. Childhood family SES is related to a myriad of life experiences [2, 4, 6]. Lower family SES is linked to increased food scarcity, reduced access to food with high nutritious quality, and more limited health care [7, 8]. Children from lower SES backgrounds are more likely to live in lower-quality residential spaces, with elevated risk for exposure to neurotoxins like lead and indoor air pollutants, residential crowding, and poor housing maintenance [8–10]. Children from lower SES backgrounds are also more likely to experience adverse neighborhood conditions, such as excessive noise and exposure to hazardous wastes [11, 12]. Furthermore, children from lower SES backgrounds are less likely to have access to well-maintained child-care facilities, schools, and recreational amenities [10, 13]. Nutrition, health resources, and the physical quality of settings where children live and learn have been linked to child outcomes directly, as well as indirectly through their associations with the well-being of family members, teachers, and peers [10, 14, 15].

In addition to the material and physical characteristics of children’s daily environments, specific family SES indicators have also been associated with the psychosocial dynamics in children’s lives, especially through parenting behaviors [16–18]. Low parental income and increased parental financial stress have been linked to disadvantageous child outcomes through increased parental distress and disrupted parenting [19]. In addition, parent educational attainment has been linked to parental beliefs and expectations about child development and what children would need to be successful, which in turn predict the extent to which parents seek cognitively stimulating activities inside and outside of the home environment [20]. It has been proposed that childhood SES may relate to brain development through the unique, cumulative, and interactive effects of these psychosocial factors, especially via changes in the neurobiology of stress regulation and via neurodevelopmental changes afforded by cognitive stimulation in children’s daily lives [2, 8, 21].

### Neurodevelopment in the context of childhood family SES

There is converging evidence that links family SES to neurodevelopment in childhood and adolescence [1, 22, 23]. SES-related differences have been reported for the development of broad brain structures, including brain volume, gray matter density, total surface area, mean cortical thickness, and white matter volume in children and adolescents [24–27]. SES-related differences have also been observed in the structure and functioning within specific brain regions supporting cognitive control, memory, language, and reading acquisition [23, 28–30], as well as the functional connectivity within and between brain networks, including the frontoparietal, sensorimotor, and the default mode networks [31–33].

Childhood family SES has also been linked to the electrophysiology of the developing brain [34, 35]. Lower parental educational attainment and lower family income-to-needs ratio have been associated with alterations in neural responses in children, such as larger neural responses to sounds appearing in a distracting story [36, 37], smaller neural responses to novel stimuli [38], and attenuated neural responses to targets in tasks of cognitive control [39, 40]. Together, these findings provide corroborating evidence that childhood family SES is associated with neurodevelopment in childhood.

A few longitudinal neuroimaging studies raised the possibility that the links between childhood family SES and brain structures and functions may persist into adulthood. For example, lower family income-to-needs ratio in childhood was associated with less white matter organization in the frontolimbic and white matter association tracts [41] and altered neural responses to stimuli with negative emotional valence [42]. Furthermore, lower average family income-to-needs ratio assessed across multiple time points from childhood to adolescence was linked to less cortical thickness and surface area in brain regions involved in a wide array of functions in adulthood [43]. However, it remains unclear whether other indicators of childhood family SES, especially parent educational attainment and subjective social status, which have distinct links with developmental outcomes [5, 20], relate to brain functions in adulthood.

### Capturing childhood family SES retrospectively

Undoubtedly, the most accurate way of investigating the links between childhood family SES and adult brain functioning is a longitudinal design that follows individuals from childhood through adulthood. Although some researchers have successfully done so [41–43], longitudinal studies spanning a decade, or more, are rare due to the extensive resources required. In the absence of longitudinal studies, and despite its limitations, an alternative approach is to assess childhood family SES by retrospectively asking adults about their childhood experiences [44, 45].

Family SES is most commonly assessed in childhood and adolescence by parent reports of parent educational attainment, occupation, and family income [4]. However, it may be harder for adults to accurately report on their childhood family income or wealth. To address this issue, similar to what is done by developmental researchers when access to parent reports is limited and youth reports of household income and wealth may not be accurate [4], childhood parent educational attainment may be used as an objective proxy for childhood family SES.

Another approach for assessing family SES is asking individuals about their subjective social standing in a particular sociohistorical context [4, 46]. Subjective social status is most commonly measured by having respondents rank themselves on a ladder of status, which places people who are the worst off, those who have the least money, the least education, and the worst jobs or no jobs at the bottom [47]. Subjective SES generally has weak to moderate associations with objective SES indicators [48] and may capture not only economic circumstances, but also relative judgments of social status based on power, control, social influence, and standing in a community and society [4, 49].

It has been argued that lower subjective social status may be linked to poorer physical and mental health outcomes, not only because it is related to deprivation from economic resources, but also because it may be related to recurring negative emotions that are elicited by upward social comparisons and relative deprivation and associated alterations in stress regulation systems [46, 49]. Several studies linked concurrent subjective family SES to physical and mental health outcomes and school achievement in adolescence [5, 50, 51]. In adults, both retrospective reports of childhood and adolescence family SES and concurrent subjective social status were associated with neural responses in situations with overt socioemotional and interpersonal salience, including when adults were asked to make social comparisons, were presented with stimuli with negative emotional valence such as threatening faces, and were given negative feedback [44, 52–54]. It remains to be investigated whether the links between subjective family SES in childhood and brain functioning in adulthood would extend to fundamental brain functions supporting perception and cognition in daily life, even in situations without overt socioemotional valence.

### Present study

The main goal of the present study was to investigate the links between objective and subjective indicators of childhood family SES and fundamental brain functions supporting perception and cognition in young adults. We used the highest parent educational attainment in childhood as a proxy for objective childhood family SES because we reasoned that parent educational attainment may be more robust to inaccuracies in recall compared to other objective indicators such as family income or wealth in childhood. In addition, adults reported on their subjective evaluations of their childhood family SES. To index brain functions supporting perception and cognition, we recorded ERPs from young adults (N = 86) during a passive auditory oddball and an active visual oddball task, which were optimized to capture two well-established and widely used event-related potentials (ERP) components—the Mismatch Negativity (MMN) and P3b respectively [55].

MMN is a neural index of change detection that occurs after an echoic memory trace is formed and is elicited once deviance in the auditory context is detected, even in the absence of attention [56, 57]. In neurotypical adults, MMN is observed as a greater negative deflection for rare versus frequent stimuli over the frontocentral electrode sites between 125 and 250 ms post-stimulus onset [55, 58]. Previous research on the links between childhood family SES and MMN is scarce. However, a limited number of studies demonstrated that MMN could be altered with training among neurotypical children and adults [59, 60]. These findings suggest that MMN can be modified by experiences. Given that childhood family SES is related to childhood auditory experiences at and outside of home [10, 20] and that supportive experiences, such as trainings have been linked to larger MMN responses [59, 60], we reasoned that higher objective childhood family SES would be associated with larger (more negative) MMN responses.

P3b, which is a subcomponent of the P3 ERP component, is considered to index brain functions that support cognitive control and are involved in the detection of rare targets, allocation of attentional resources to the target, stimulus evaluation, and updating neural representations associated with memory operations [61, 62]. In neurotypical adults, P3b is observed as a robust positive voltage deflection for rare targets compared to frequent stimuli over parietal electrode sites between 300–600 ms [55]. Specific indicators of childhood family SES, in particular, higher maternal education [40] and higher family income [63] have been associated with larger P3 amplitudes in children. Building on these findings, we expected higher objective childhood family SES to be related to larger (more positive) P3b responses. As subjective evaluations of childhood family SES may capture SES-related life experiences above and beyond those related to parent educational attainment, such as stress resulting from limited financial resources or social comparisons [4, 49], we also hypothesized that higher childhood subjective family SES would be associated with larger neural responses for MMN and P3b, even after taking childhood parent educational attainment into account.

## Method

### Participants

Participants were recruited via flyers posted on a university campus and at community centers in the Western United States. The age range for participant recruitment was set to be 18 to 30 years to make our sample comparable in age with the sample in the ERP CORE study from which we adopted our tasks, data processing pipeline, and ERP scoring methods [55]. The inclusion criteria for recruitment were as follows: normal or corrected-to-normal vision, no hearing issues, no history of brain injuries or neurological disorders, and no current use of medications that could alter brain functioning. The study protocol was approved by the Institutional Review Board of the University of California Merced. All individuals gave written consent to participate in the study. All participants received cash for their participation. The data collection for the study started on February 23^rd^, 2023, and ended on June 16^th^, 2023.

An *a priori* power analysis was conducted in G*Power to determine an adequate sample size before data collection had started. With two predictors (objective and subjective childhood family SES), an alpha level of .05, an effect size of .15, and a minimum power established at .80, we determined that a minimum sample size of 68 would be needed per ERP component. To account for a 5% data loss rate in adult studies, we aimed for a minimum sample size of 72.

Participants were excluded from the analytical sample if they completed the sociodemographic questionnaire incorrectly (i.e., reported on the educational attainment of adults with whom they currently live with, such as roommates, instead of their parents/legal guardians, *n* = 5) or had poor ERP data quality for both ERP tasks (*n* = 1). Due to experimenter error, participant performance, or ERP data quality, we had usable data for only one of the two ERP tasks for some participants. To maximize our sample size, we included all participants who had at least one usable ERP task in the present study. The participants who had data from only one ERP task (*n* = 24) did not differ from participants who had data from both tasks (*n* = 62) in terms of age, gender, education level, highest childhood parent educational attainment, and subjective childhood family SES (all *p*s > .17). Table A in S1 Text includes the descriptive statistics for these sociodemographic variables for each group. Table B in S1 Text provides a summary of the independent samples t-tests comparing the sociodemographic characteristics of the participants who had data from only one ERP task to participants who had data from both tasks.

The final analytical sample consisted of 86 adults between the ages of 18 and 30 years (M = 21.85, *SD =* 2.74). When asked to report their gender, 55% of the participants chose female, 45% chose male, and no other genders were reported. Participants reported their race/ethnicity as follows: 57% Hispanic or Latino/x, 16.3% Asian/Asian American, 16.3% selected multiple categories, 9.3% White/European American, and 1.1% not reported. All participants reported having completed at least high school, and 12% had an associate degree, 21% had a bachelor’s degree, and 3.5% had a master’s degree (see Fig A in S1 Text for participant education frequencies). The childhood parent educational attainment levels of the participants were as follows: 28% less than a high school diploma, 20% high school diploma, 14% some college but no degree completed, 6% associate degree, 14% bachelor’s degree, 12% master’s degree, and 6% professional or doctorate degree.

### Childhood family SES

The SES questionnaires were administered after the completion of the ERP tasks to avoid prompting the participants to think about their childhood SES before or during the ERP tasks. To obtain retrospective reports of objective childhood SES backgrounds and subjective social status, participants were asked to think back to when they were 10 years old to answer the questions.

#### Objective childhood family SES

Parent education was used as a proxy for objective childhood family SES. Participants were instructed to report the highest level of education attained by at least one parent/legal guardian (e.g., mother, father, grandparent, stepparent, etc.). The educational attainment categories were similar to those used in the Survey of Income and Program Participation [64]. These categorical labels were recoded as years of education (see Appendix A in S1 Text for the childhood family SES questionnaire and coding schema). Participants were given the option to report on the education levels of up to 4 adults. To reflect the diverse family compositions of the participants (e.g., uncle as legal guardian in childhood), instead of maternal education, the highest education level completed by a parent/legal guardian (hereafter referred to as parent) was used (see Fig B in S1 Text for childhood parent educational attainment frequencies).

#### Subjective family SES in childhood

The MacArthur Scale of Subjective Social Status (Adler et al. 2000) was adapted to measure subjective family SES in childhood. Participants were instructed to indicate where they would place their family in comparison to American society on a ladder, with steps numbered 1 to 10, with 1 being at the bottom of the ladder and 10 being at the top (see Fig C in S1 Text for childhood subjective family SES frequency distribution).

### Oddball tasks

The passive auditory oddball and the active visual oddball tasks were adapted from ERP CORE, a set of paradigms designed to optimally capture widely studied ERP components [55]. The tasks were presented using the Presentation (Neurobehavioral Systems) experiment software. The visual stimuli were presented on a Dell LCD monitor with a resolution of 1280 x 1024, a refresh rate of 60 Hz, and a viewing distance of 95 cm. Participants were offered to take a break in between tasks.

#### Passive auditory oddball

The original ERP CORE passive auditory oddball task [55] included 1000 trials (approximately 10 minutes long). To adapt the task for use in future studies with a wider age range, including young children, we reduced the duration of the task to approximately 3.5 minutes and changed the video that accompanies the task to a more engaging child-friendly cartoon, “Pingu” the penguin. The other task characteristics were kept consistent with ERP CORE. The details of why and how the task was modified can be found in the Supporting Information (S1 Text). Participants were told they would hear a series of sounds and were instructed to ignore the sounds while watching the silent video. Participants were presented with 350 trials. A frequent tone was presented at 80 dB for 100 ms in 80% of trials. A rare tone was presented at 70 dB for 100 ms in 20% of trials. The auditory stimuli were presented with noise-canceling headphones. Interstimulus intervals jittered between 450–550 ms. The task began with 15 frequent tones to allow for the auditory system to habituate to the standard tone.

#### Active visual oddball

This task was identical to the ERP CORE version, except for how behavioral responses were collected (we used a game controller instead of a keyboard). The visual stimuli were presented on a medium gray background (x = 0.35, y = 0.36, 25.9 cd/m^2^). A white fixation point (0.15° visual angle) was presented at the center of the screen, and participants were instructed to maintain fixation on this point throughout the task. On each trial, a single capitalized letter of five letters (e.g., A, B, C, D, or E) in the Geneva font appeared on the display, covering a visual angle of 2.5 x 2.5° for 200 ms. Sequential stimuli were presented over the fixation point and separated by an interstimulus interval of 1200–1400 milliseconds. Participants completed 200 trials, divided into 5 blocks. In each block, 1 letter was designated the target stimulus (rare: 20% probability), and the other 4 were designated as non-targets (frequent: 80% probability). Participants were instructed to indicate whether the stimulus presented was a target, using an “up” button press or a non-target letter using a “down” button press on the game controller for that given block. Each of the five letters served as a target in one block of the experiment and as a non-target in the other four blocks, with the order of blocks randomized across participants.

### Electroencephalogram (EEG) recording

**Fig 1:**
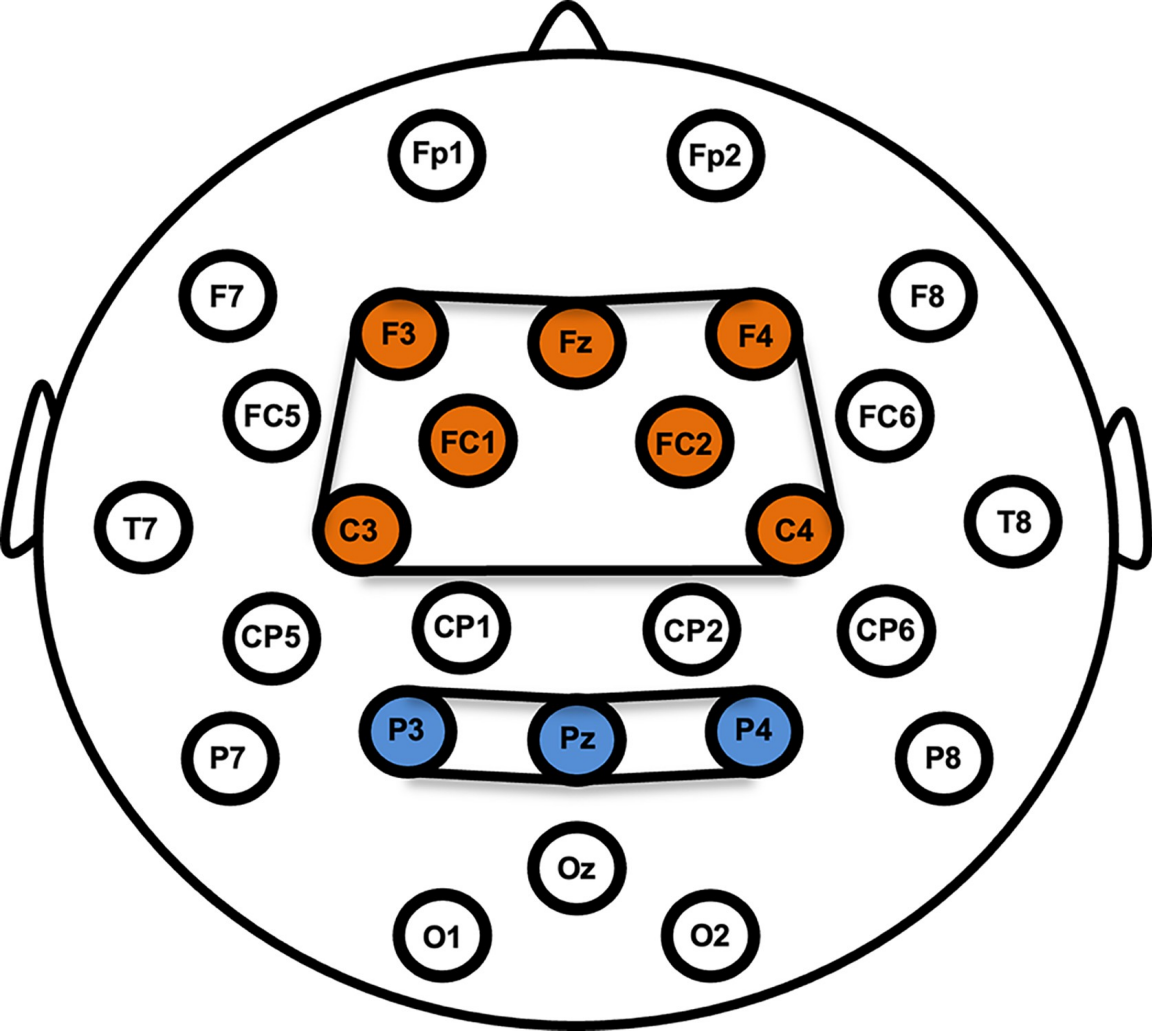
Configuration of the scalp electrodes. Channels shaded in orange are included in the frontocentral cluster used for MMN analyses. Channels shaded in blue are included in the posterior cluster used for P3b analyses.

EEG was recorded with Brain Products actiCHamp Plus, using BrainVision Recorder Version 1.25.0101, and collected with an actiCAP slim active electrode system, mounted on elastic snap caps [65]. The ground electrode was placed at FPz. From the 32-channel electrode bundle, 2 electrodes were repurposed and placed on the mastoid bones behind the left and right ears for offline re-referencing. Three additional electrodes were repurposed to record electrooculogram (EOG). The vertical EOG (VEOG) electrode was placed below the right eye and the horizontal EOG (HEOG) electrodes were placed lateral to each eye’s external canthus. The remaining 27 electrodes were used as scalp electrodes, mounted in accordance with the international 10/20 system (see Fig 1 for the electrode configuration). EEG was sampled at 500 Hz and referenced to Cz. The stimulus presentation delays in the monitor and the headphones were assessed with StimTrak [65]. Both the visual and auditory stimuli were delayed by approximately 20 ms.

### EEG signal processing and averaging

EEG signal processing and averaging were conducted in MATLAB using customized EEGLAB [66] and ERPLAB [67] scripts, as well as scripts adapted from ERP CORE [55] and ICLabel [68]. The data processing and analysis modifications were made to have a pipeline that can be easily adapted for use with a wider age group and was previously used with young children [40, 69]. All scripts can be found on Open Science Framework (OSF): https://osf.io/43h75/.

EEG data were re-referenced to the arithmetic average of the left and right mastoids. To detect ocular artifacts more easily, bipolar eye channels were created as follows: the vertical bipolar eye channel was computed by subtracting FP2 from the right VEOG, and the horizontal bipolar eye channel was computed by subtracting the left HEOG from the right HEOG. EEG data were band-pass filtered with the EEGLAB default finite impulse response (FIR) filter between 0.1 to 40 Hz (-6 dB cutoff frequency). This filter was chosen to be consistent with previous and ongoing research with children [40, 69].

EEG artifacts were removed using a combination of independent component analysis (ICA) and the rejection of trials with residual artifacts. To prepare data for ICA, we removed recording periods with no event codes (defined as no event codes for 6000 ms or longer, with a buffer of 3000 ms before and after any event codes). To detect bad channels that would cause unnecessary data loss, we applied the ERPLAB moving window peak-to-peak threshold algorithm across a 500 ms window, moving in 50 ms increments, with a +/- 300 μV threshold to all the scalp channels. Except for FP1 and FP2, any scalp channel that resulted in excessive data loss (defined as more than 10% overall data loss and ≥ 3.29 *SD* data loss compared to other scalp channels) was excluded from pre-ICA artifact rejection. Then, data segments with extreme artifacts were rejected from the continuous data with the ERPLAB moving window peak-to-peak threshold algorithm (across a 500 ms window, moving at 50 ms increments, with a +/- 300 μV threshold) applied to all remaining scalp channels, excluding FP1 and FP2.

ICA was applied to all channels, except the bipolar eye channels. The computed ICA weights were applied to the preprocessed data files (i.e., before pre-ICA cleaning). Artifact correction was conducted by removing components that were selected as “eye” components if ICLabel classified them as “eye” with at least 80% confidence and as “brain” with less than 5% confidence. These criteria were selected based on a systematic comparison of different confidence thresholds for young adults.

Before epoching, to account for the equipment presentation delays described above, the stimulus event codes were shifted in time for 20 ms. Consistent with the ERP CORE parameters, ICA-corrected data were epoched -200 to 800 ms relative to stimulus onset, and the -200 to 0 ms pre-stimulus onset was used for baseline correction. To remove remaining eye artifacts, ICA-corrected bipolar HEOG and VEOG channels were computed. To avoid unnecessary data loss due to a bad channel, first, a simple voltage threshold algorithm with a +/- 200 μV threshold was applied to all the scalp channels of analytic interest. The channel exclusion criteria were the loss of more than 10% of trials and a data loss rate of at least 3.29 SD above the other channels. In the auditory oddball task, 4 participants had 1 “bad channel” and these scalp channels were excluded from further artifact rejection and analyses. In the visual oddball task, no channels of interest were detected to be excluded.

The final artifact rejection included the following steps: To detect artifacts in scalp channels of interest, we applied a simple voltage threshold algorithm with a +/- 200 μV threshold and a moving peak-to-peak window algorithm moving at 100 ms increments, with a 125 μV threshold to each epoch. To remove residual blinks and saccades, a moving peak-to-peak window algorithm was applied to the ICA-corrected VEOG (across a 200 ms window, moving at 50 ms increments, with a 150 μV threshold), and a step-like algorithm was applied to the ICA-corrected HEOG (across a 100 ms window, moving at 10 ms increments, with a 64 μV threshold). The following steps were added for the visual oddball task: To reject trials where a blink occurred too close to when the visual stimuli were presented, we applied a moving peak-to-peak window algorithm to the uncorrected VEOG (between -25 to 225 ms with respect to stimulus onset, moving at 10 ms increments, with a 150 μV threshold). To reject trials where a saccade occurred too close to when the visual stimuli were presented, we applied a step-like algorithm to the uncorrected HEOG (between -50 to 250 ms with respect to stimulus onset, moving at 10 ms increments, with a 32 μV threshold). Consistent with ERP CORE, in the visual oddball task, trials with incorrect behavioral responses and trials with responses faster than 200 ms and slower than 1000 ms were also excluded from analyses.

After artifact rejection, individual ERP plots were visually inspected for data quality. In addition, analytic standardized measurement error (aSME) was computed for observed waveforms as an objective metric of data quality [70] and used to check for data quality in channels of interest across participants. In the auditory oddball, 1 participant was excluded for being an aSME outlier and not showing discernable auditory evoked potentials. In the visual oddball, 2 participants were excluded for being aSME outliers and not showing discernable visual evoked potentials.

### ERP amplitude and data quality scores

In ERP CORE [55], single channels were selected for each component of interest (e.g., FCz for MMN and Pz for P3b). However, in a follow-up study, analyses conducted with the ERP CORE data, as well as Monte Carlo simulations, revealed that the ERPs obtained from multi-site clusters had as good or better data quality compared to ERPs obtained from a single channel of interest for all ERP components, including MMN and P3b [71]. Therefore, we inspected the grand average plots and selected multiple channels in which ERP components of interest were similarly observable. We created multi-site channel clusters for ERP analyses as follows: Fz, F3, F4, FC1, FC2, C3, C4 for MMN; Pz, P3, and P4 for P3b. Figs F and H in S1 Text show the grand average plots for the MMN and P3b channel clusters respectively, and Figs G and I in S1 Text Fig show the grand average plots for MMN and P3b over representative scalp channels respectively.

ERP time-window mean amplitude scores (hereafter referred to as mean amplitude) were extracted for MMN between 125 and 225 ms in the auditory oddball task, and for P3b between 300 and 600 ms in the visual oddball task, consistent with the time windows recommended in ERP CORE [55]. Given that difference waves eliminate concurrent neural processes across conditions [72], we computed difference waves (rare minus frequent) to isolate the experimental effects in the auditory and visual oddball tasks. To provide an objective data quality metric for the MMN and P3b difference waves, bootstrapped standardized measurement error (bSME) scores [70] were computed.

### Behavioral performance

A discriminability index (*d*′) was calculated as a measure of behavioral performance in the active visual oddball task as follows: *d*′ = *Z*(Correct/Hit)—*Z* (Incorrect/False Alarm). Higher values of *d*′ indicate a greater ability to distinguish signals from noise, corresponding to better overall task performance [73].

### Data analytic plan

Hierarchical regression analyses were conducted separately for MMN and P3b mean amplitudes. In each regression analysis, the highest parent education level in childhood was entered in Step 1. The subjective childhood family SES was added to the model in Step 2. All artifact-free trials were used in MMN analyses since there were no response demands. Only correct trials were included in P3b analyses. All analyses were performed in Jamovi (Version 2.3.28). The data file used in the analyses and the accompanying data dictionary are available on OSF: https://osf.io/43h75/.

## Results

First, preliminary analyses were conducted to check for univariate outliers for all variables of interest. Any score above or below 3.29 *SD* was considered an outlier, because in relatively large samples, 99.9% of *z*-scores lie between -3.29 and 3.29, and the likelihood of an absolute *z*-score value of 3.29 or greater being sampled from the population of interest is high unlikely [74]. There were no outliers for childhood parent education levels, subjective childhood family SES, or the mean amplitude and SME values for MMN or P3b. However, we detected 1 outlier for behavioral performance (*d*’ *z*-score less than -3.29) in the visual oddball task. Therefore, all visual oddball analyses (*d*’ and P3b) were conducted with and without this participant. Because the strength and direction of the results were consistent with and without this outlier, to reflect the true range of the scores, the results reported here include this outlier.

Descriptive statistics for participant age, participant education level, highest childhood parent educational attainment, subjective childhood family SES, behavioral performance in the visual oddball task (*d*-prime), and MMN and P3b difference wave mean amplitude and bSME values are reported in Table 1. The descriptive statistics for the number of artifact-free ERP trials, ERP mean amplitudes, and aSME values for frequent and rare conditions in the auditory and visual oddball tasks are reported in Table C in S1 Text.

**Table 1:** Descriptive statistics for participant sociodemographic characteristics, ERP mean amplitude and data quality scores for MMN and P3b difference waves, and behavioral performance in the visual oddball task.

Variables	N	Mean	*SD*	Min	Max
Age	86	21.84	2.74	18.58	30.12
Participant education	86	13.51	1.7	12	18
Childhood parent education	86	12.52	4.26	4	20
Childhood subjective SES	86	4.89	2.08	1	10
MMN mean amplitude (μV)	73	-2.18	1.52	-5.83	1.44
MMN bSME	73	1.16	.21	.53	1.69
P3b mean amplitude (μV)	75	5.94	3.36	-1.04	15.93
P3b bSME	75	1.72	.41	1.08	3.06
*d*’	75	3.42	.70	1.89	5.15

*Note*. Childhood parent education: Highest parent educational attainment in years; Childhood subjective family SES is on a scale of 1–10, with higher values showing higher subjective family SES; *d*’: index of behavioral performance in the visual oddball task, with higher values denoting better task performance; bSME: bootstrapped standardized measurement error, with lower values corresponding to higher ERP data quality.

Zero-order correlations for all variables of interest are reported in Table 2. We conducted preliminary analyses to determine if there were any control variables we should take into consideration. Participant age and gender were not related to any outcomes of interest. In addition, childhood SES variables (parent education and subjective social status) were not related to ERP data quality, implying that any SES-related differences in ERP amplitudes would not be confounded by SES-related differences in data quality.

**Table 2:** Zero-order correlations for variables of interest.

Variables	1	2	3	4	5	6	7	8	9	10
1. Age	-	-	-	-	-	-	-	-	-	-
2. Gender	-.15	-	-	-	-	-	-	-	-	-
3. Participant education	.71*	-.08	-	-	-	-	-	-	-	-
4. Childhood parent education	-.30*	.28*	-0.18	-	-	-	-	-	-	-
5. Childhood subjective SES	-.39*	.11	-.23*	.41*	-	-	-	-	-	-
6. MMN mean amplitude	.03	-.10	-.01	-.13	.05	-	-	-	-	-
7. MMN bSME	-.11	-.15	-.02	.05	-.04	-.09	-	-	-	-
8. P3b mean amplitude	-.19	.06	-.15	.26*	-.02	-.26*	.15	-	-	-
9. P3b bSME	-.05	-.12	-.09	.03	.03	-.11	.31*	.13	-	-
10. d’	-.03	-.17	.01	-.11	.04	-.01	-.19	-0.01	-.04	-

Note

* *p* < .05; Gender: 0 = female; 1 = male (no other genders were reported); Childhood parent education: Highest parent educational attainment in years; Childhood subjective family SES is on a scale of 1–10, with higher values showing higher subjective family SES; *d*’: index of behavioral performance in the visual oddball task, with higher values denoting better task performance; bSME: bootstrapped standardized measurement error, with lower values corresponding to higher ERP data quality.

There was a negative correlation between MMN and P3b amplitudes. Taking the opposite polarity of these ERP components into account, participants who had larger (more negative) MMN mean amplitudes also had larger (more positive) P3b mean amplitudes. There was also a positive correlation between MMN data quality and P3b data quality. Participants who had higher data quality in the auditory oddball task also had higher data quality in the visual oddball task.

**Fig 2:**
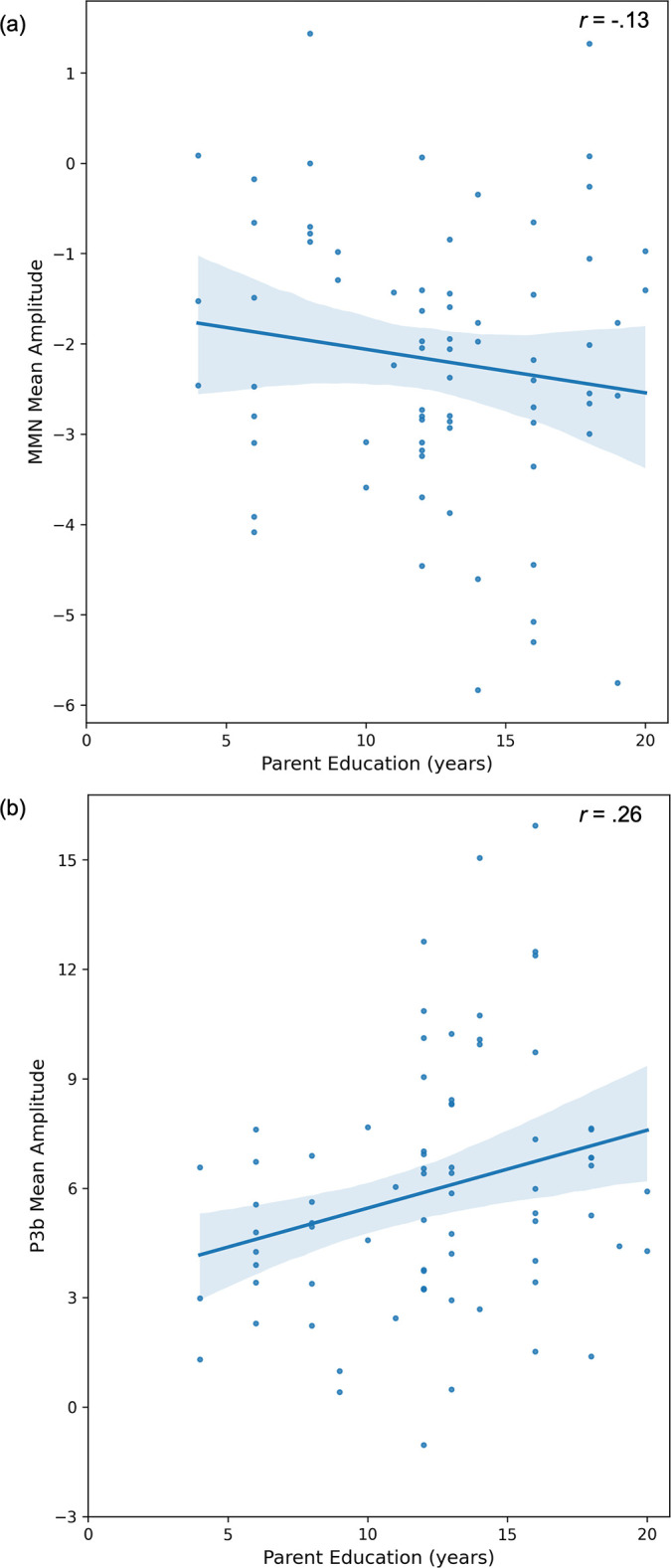
Scatter plot for the zero-order correlations between childhood parent educational attainment and ERP mean amplitude scores for MMN (a) and P3b (b).

**Fig 3:**
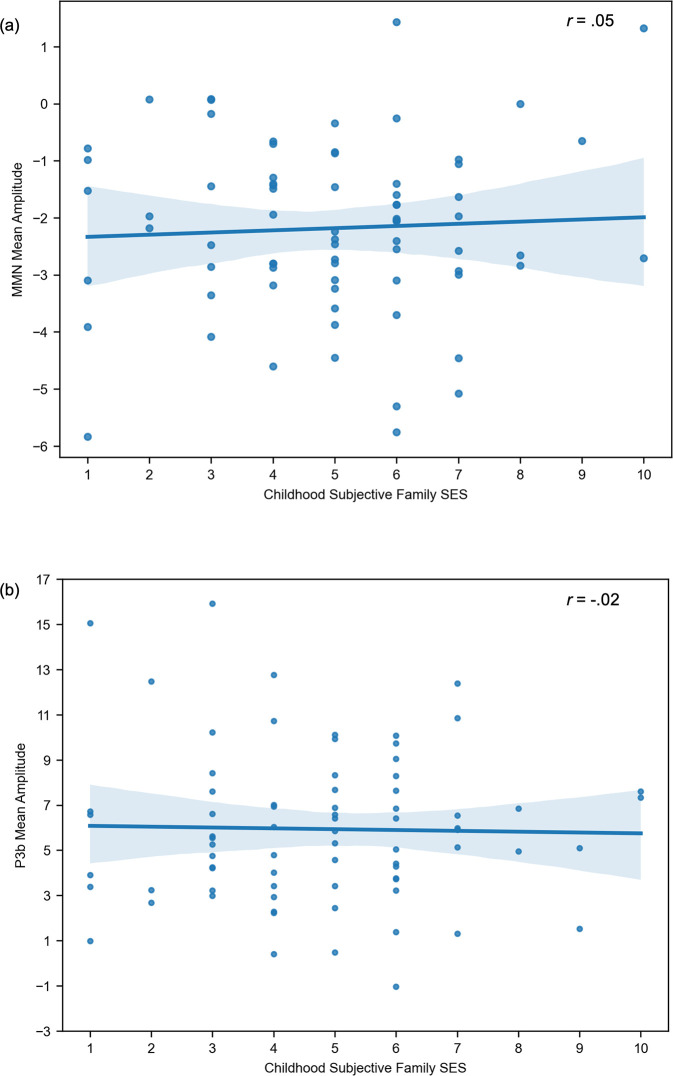
Scatter plot for the zero-order correlations between childhood subjective family SES and ERP mean amplitude scores for MMN (a) and P3b (b).

A post-hoc exploratory data analysis showed no link between P3b mean amplitude and behavioral performance in the visual oddball task. Childhood SES measures also were not related to behavioral performance in the visual oddball task. The zero-order correlations between childhood SES indicators and ERP mean amplitude scores for MMN and P3b are depicted in Figs 2 and 3.

The statistics for the hierarchical regressions are reported in Table 3. The first hierarchical regression was conducted for MMN. There was no link between childhood parent educational attainment and MMN mean amplitude. The addition of subjective childhood SES did not significantly contribute to the regression model. Overall, this regression analysis did not reveal any associations between childhood SES and MMN amplitude.

**Table 3:** Summary of hierarchical linear regression analyses for MMN and P3b mean amplitudes.

Variables	*B*	*SE B*	*β*	*p*	*R* ^*2*^
MMN					
Step 1					.02
Parent education	-.05	.04	-.13	.27	
Step 2					.03
Parent education	-.07	.05	-.20	.13	
Subjective SES	.11	.10	.15	.27	
P3b					
Step 1					.07
Parent education	.21	.09	.87	.02	
Step 2					.09
Parent education	.27	.10	.33	.01	
Subjective SES	-.26	.20	-.16	.20	

*Note*. Childhood parent education: Highest parent educational attainment in years; Childhood subjective family SES is on a scale of 1–10, with higher values showing higher subjective family SES.

**Fig 4:**
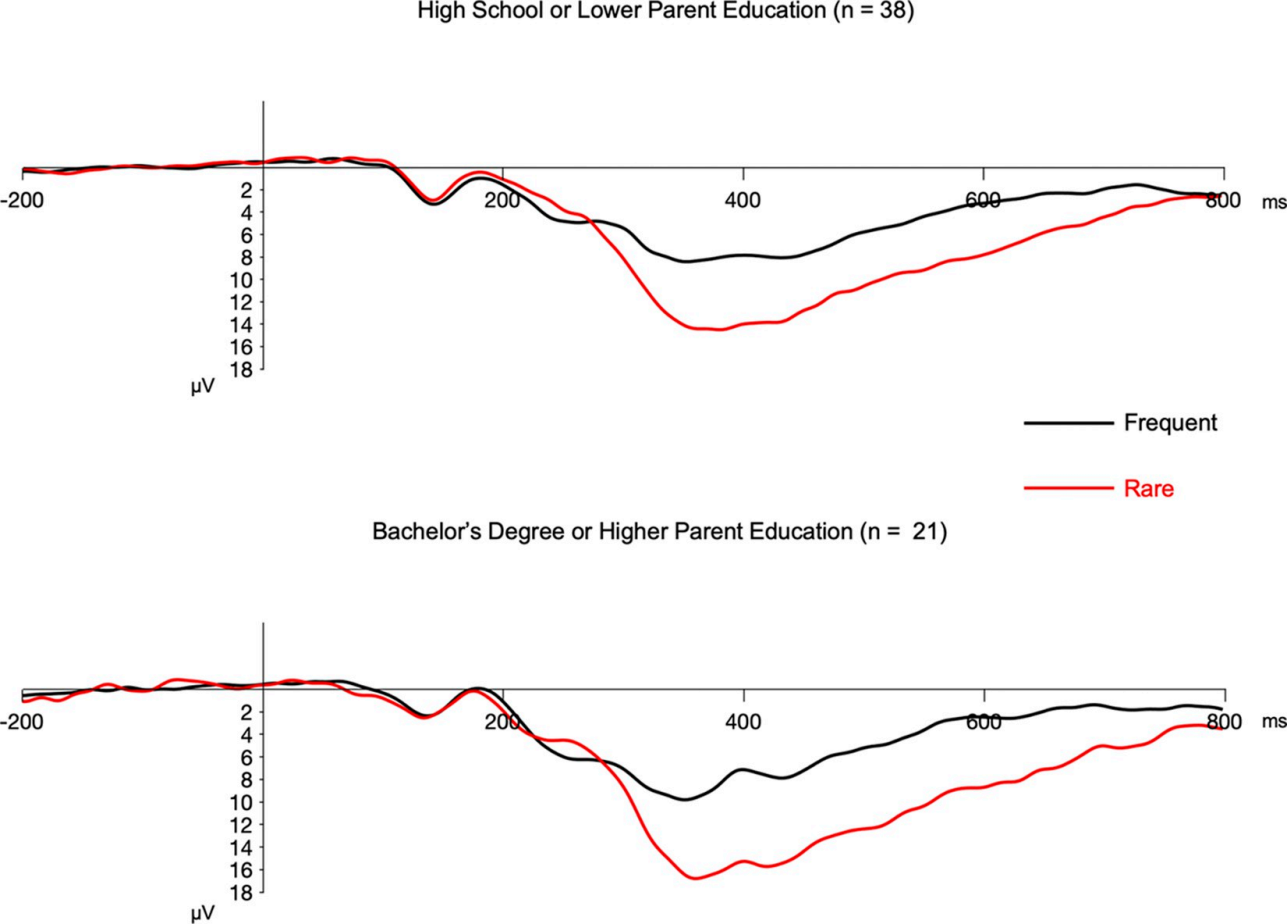
Grand average ERP plots for frequent (black waveform) and rare (red waveform) trials over the parietal channel cluster used for measuring P3b in the visual oddball task. By convention, negative is plotted upward. P3b was measured between 300–600 ms post-stimulus onset. This figure is only created for visual illustration purposes and includes only adults who reported high school diploma or less for their childhood parent educational attainment (left; *n* = 38) versus adults who reported bachelor’s degree or above for their childhood parent educational attainment (right; *n* = 21). The grand average was not plotted for the participants who reported some college classes without any degree completion for their childhood parent educational attainment since the sample size of this group was very small (*n* = 6).

The second hierarchical regression was conducted for P3b. As expected, childhood parent educational attainment was a significant predictor of the P3b amplitude. Higher childhood parent educational attainment was linked to larger (more positive) P3b amplitude. For visual illustration purposes, Fig 4 depicts P3b in adults who reported high school diploma or less for their childhood parent educational attainment versus adults who reported bachelor’s degree or above for their childhood parent educational attainment. The addition of subjective childhood SES did not significantly contribute to the regression model. Therefore, contrary to our hypothesis, after taking childhood parent educational attainment into account, subjective childhood SES was not linked to P3b amplitude.

## Discussion

The present study examined to what extent objective and subjective indicators of childhood family SES related to two well-established electrophysiological indices of brain functioning in adulthood—the MMN and P3b ERP components. Objective childhood family SES, as proxied by the highest parent educational attainment in childhood, was linked to the magnitude of P3b in young adults. Specifically, higher parent educational attainment in childhood was associated with larger (more positive in amplitude) P3b responses. In contrast, there was no association between parent educational attainment and the magnitude of the MMN component. These findings imply that the links between childhood SES and brain functioning may persist into adulthood, specifically pertaining to brain functions that support cognitive control rather than fundamental brain functions that may precede but do not require the engagement of cognitive control. Adult reports of subjective family SES during childhood were not related to the magnitude of either P3b or MMN. These results highlight that, when using retrospective accounts of childhood family SES, objective and subjective reports likely proxy different childhood experiences with distinct and enduring ties to certain neurodevelopmental outcomes, and that such associations may not exist or persist into adulthood for various other aspects of neurodevelopment.

In children, family SES has been linked to specific ERP components, especially those that appear relatively later in timing and are involved in cognitive control, rather than ERP components that occur earlier in timing and index more automatic neural responses [36, 40, 63]. Similarly, we found that childhood parent educational attainment was linked to the neural index of cognitive control, the P3b, and not the earlier and more automatically appearing MMN. Although previous research linked musical and phonetic training to changes in MMN amplitude in neurotypical children and adults [59, 60], implying that MMN is sensitive to environmental influences, we did not find any links between childhood family SES and MMN. It is possible that environmental influences on neural functions indexed by MMN may be more short-lived compared to other brain functions. As adults experience various auditory environments from childhood through adulthood, brain functions indexed by MMN may be adapting to these new auditory environments rapidly.

In previous research with children, lower levels of parent educational attainment and family income-to-needs ratio were associated with attenuated neural responses in tasks that engage cognitive control skills, such as selective attention and inhibitory control [37, 38, 75]. In addition, as it directly pertains to our findings, previous research linked lower parent education levels and income-to-needs ratio to smaller P3b magnitude in children [40, 63]. Extending these findings into adulthood, we also found lower parent educational attainment during childhood to be associated with smaller P3b in adults in a visual oddball task that requires cognitive control skills, such as sustaining attention to targets, engaging working memory to keep rules in mind, memory updating, and inhibiting automatic but task-irrelevant responses. This finding implies that the links between childhood family SES and brain functions supporting cognitive control may persist into adulthood.

In the present study, we used childhood parent educational attainment as an objective indicator of childhood family SES. This indicator might have served as a proxy for various environmental, material, and psychosocial factors that have been associated with childhood family SES [2, 10, 16]. However, parent education may also be a unique predictor of adult brain functioning. Higher parent educational attainment has been linked to greater parental investment in cognitively stimulating activities in and outside of the home [20, 76], even in families with lower income [77] or after taking family income into consideration [76]. In both human and non-human animal studies, as well as computational modeling studies, environmental enrichment and cognitive stimulation have been linked to alterations in brain structures and functions [21, 76, 78]. To speculate, higher parent educational attainment may uniquely contribute to adult brain functioning through environmental enrichment and cognitive stimulation in childhood.

Previous research suggested that parent education and family income in childhood may contribute to developmental outcomes through distinct mechanisms, such as higher parent education levels being linked to advantageous outcomes through increased exposure to cognitively stimulating activities in childhood, or increased financial stress being linked to disadvantageous outcomes through increased parenting stress and disrupted parenting [17–20]. Therefore, it is plausible that parent education and family income in childhood may have unique associations with adult brain functioning. The retrospective design of our study precluded us from addressing this important question as we did not have information about the childhood family income or wealth of our participants. Based on the research showing that adolescents could provide accurate information about their parental education, but not their family income [4], we reasoned that when asked to think back about their childhood, young adults would also find it difficult to provide accurate information about their family income. Indeed, in a pilot assessment we conducted, none of the adults could report on their childhood family income with confidence, whereas they could report on their parent education levels and provide a subjective evaluation of their family social standing. Accordingly, we chose not to include an item about childhood family income in our questionnaire. Although it may not be feasible to obtain retrospective accounts of family childhood income from adults accurately, accounts of family economic hardship during childhood may act as proxy for family income-to-needs ratio and wealth and relate to adult brain functioning. For example, in a recent study with young adults, retrospective reports on the extent of family material hardship encountered before 18 years of age in paying for food, clothing, housing, and medical care was associated with patterns of connectivity in the frontolimbic circuitry [45]. Future research including retrospective accounts of not only parent education levels but also family financial hardship in childhood, and specific mechanisms through which these family SES indicators may be linked to neurodevelopmental outcomes, will allow us to gain a more comprehensive understanding of how childhood family SES relates to brain functioning in adulthood.

Although our primary focus was brain functioning, we also conducted a post hoc exploratory analysis to examine links between childhood SES and behavioral performance. Despite the association between parent education and P3b, we did not find any links between parent education and behavioral performance in the same task. This may be because the visual oddball task we used, which was successfully optimized to capture the P3b component in adults [55], was not optimal for capturing individual differences in behavioral performance. This may also explain why P3b was not associated with behavioral task performance in the visual oddball task.

However, it is also possible that SES-related differences in brain functions reflect adversity-mediated adaptations in neurodevelopment that allow individuals to utilize brain structures and functions differently. Accordingly, similar behavioral outcomes or distinct brain-behavior associations may be observed across individuals from diverse family SES backgrounds, without notable performance enhancements or decrements. There are a few examples of SES-related differences in brain-behavior associations in children and adolescents. For example, in a large nationally representative sample of children, lower resting-state functional coupling between lateral frontoparietal and the default mode networks related to cognitive test performance, but only in children from higher-income families [79]. In contrast, this relation was almost reversed in children raised in households defined as living in poverty. As another example, bilateral thickness of the rostrolateral prefrontal cortex positively correlated with fluid reasoning abilities of young children and adolescents with lower parental education levels, but not in children and adolescents with higher parent education levels [80]. SES-moderated brain-behavior associations have also been observed in neurodevelopmental disorders. For instance, reading disorders were explained by differences in phonological skills and associated activation in left inferior frontal and temporoparietal regions during phonological processing in children from higher SES backgrounds, whereas in children from lower SES backgrounds, reading disorders were explained by differences in rapid naming skills and associated activation in left temporoparietal and fusiform regions during orthographic processing [81]. Together, these developmental studies suggest that how and which brain functions support behavioral outcomes may depend on the family SES backgrounds of individuals.

Similarly, adults in our study who were raised in lower SES backgrounds might have relied less on the neural processes indexed by P3b, thus displaying smaller P3b responses, while relying on brain functions that we did not index in our study, to perform behaviorally at similar levels compared to their peers from higher childhood SES backgrounds. Future research with larger samples that are required for moderation analyses and using cognitively more challenging tasks may reveal differences in brain-behavior relations in adults depending on their childhood parent educational attainment. Previous research linked lower parent educational attainment to various factors that may contribute to developmental outcomes, such as less access to and investment in cognitively stimulating activities and enrichment for children, heightened parenting stress and associated mental health issues, and increased household chaos [20, 82, 83]. It remains to be investigated what mechanisms tie lower childhood parent educational attainment to brain-behavior associations that may reflect adversity-mediated adaptations in neurodevelopment from childhood to adulthood.

Contrary to our expectations, retrospective reports of childhood subjective SES were not linked to brain functioning in adulthood. It is possible that children’s own subjective experiences of social status may not have enduring links with adult brain functioning. However, it is also plausible that retrospective subjective accounts of childhood family SES may be prone to inaccurate recall, or reinterpretation of childhood experiences in adulthood, especially due to qualitative and quantitative changes in subjective evaluations of family SES across development. Although ladder measures of subjective social status appear to be internally consistent and reliable among children across diverse populations [84], young children tend to overestimate their subjective social status compared to their objective SES until about 10 years of age [85]. Although we asked participants to think back to when they were 10 years old, their recollections may not have captured how they had evaluated their family SES in middle childhood. Furthermore, subjective social status tends to decline with age, becomes more consistent with objective indicators of social advantage, and appears stable in adolescence and early adulthood [5, 50]. Asking adults about their childhood, adolescence, and adulthood subjective SES may reveal different patterns of results we could not capture in our study.

Another possibility is that the links between childhood subjective family SES and brain functioning may pertain to the development of brain functions not indexed by MMN or P3b. Previous research linked concurrent subjective social status to the structure and functioning of brain systems involved in social comparisons, regulation of psychosocial stress, and reappraisal of negative feedback [52–54]. Children and adolescents’ perceptions of their family social status may be more directly related to the development of the brain systems involved in socioemotional and interpersonal processes, rather than the fundamental brain functions supporting perception and cognition in situations without overt emotional and interpersonal valence.

Childhood family SES may also be linked to brain functions in adulthood indirectly through concurrent objective and subjective SES. We could not assess any such indirect paths in our study due to study design and limitations. First, the sample size we determined based on the planned regression analyses would be insufficient for conducting even exploratory tests of indirect effects. Second, there were no links between participant educational attainment and brain functioning to imply potential indirect effects. This was most likely due to our sample consisting of adults with relatively high individual education levels—despite their diverse childhood SES backgrounds, all participants in our study had completed at least high school and had taken some college courses. Future investigations are warranted to examine the direct and indirect links between objective and subjective indicators of SES in childhood, adolescence, and adulthood and neurodevelopment.

To conclude, the present study contributes to our understanding of the links between childhood family SES and brain functioning in adulthood. Our findings imply that the links between childhood parent educational attainment and brain functioning may continue into adulthood, especially for brain functions supporting cognitive control. These findings lay the groundwork for future investigations on how and why childhood family SES relates to brain functioning in adulthood and the identification of risk and protective factors for neurodevelopment from childhood to adulthood.

## Supporting information

S1 Text (DOCX)

## References

[1] Dufford AJ, Kim P, Evans GW. The impact of childhood poverty on brain health: Emerging evidence from neuroimaging across the lifespan. Int Rev Neurobiol. 2020;150:77–105. doi: 10.1016/bs.irn.2019.12.001 32204835

[2] Kim P, Evans GW, Chen E, Miller G, Seeman T. How socioeconomic disadvantages get under the skin and into the brain to influence health development across the lifespan. In: Halfon N, Forrest CB, Lerner RM, Faustman EM, editors. Handbook of Life Course Health Development. Cham (CH): Springer; 2017. 31314284

[3] Pollak SD, Wolfe BL. How developmental neuroscience can help address the problem of child poverty. Dev Psychopathol. 2020 Dec;32(5):1640–56. doi: 10.1017/S0954579420001145 33427175 PMC8346912

[4] Diemer MA, Mistry RS, Wadsworth ME, López I, Reimers F. Best practices in conceptualizing and measuring social class in psychological research. Anal Soc Issues Public Policy. 2013 Dec;13(1):77–113.

[5] Goodman E, Huang B, Schafer-Kalkhoff T, Adler NE. Perceived socioeconomic status: A new type of identity that influences adolescents’ self-rated health. J Adolesc Health Care. 2007 Nov 1;41(5):479–87. doi: 10.1016/j.jadohealth.2007.05.020 17950168 PMC2204090

[6] Cohen S, Janicki-Deverts D, Chen E, Matthews KA. Childhood socioeconomic status and adult health. Ann N Y Acad Sci. 2010 Feb;1186:37–55. doi: 10.1111/j.1749-6632.2009.05334.x 20201867

[7] Evans GW. The environment of childhood poverty. Am Psychol. 2004 Feb;59(2):77–92. doi: 10.1037/0003-066X.59.2.77 14992634

[8] Jensen SKG, Berens AE, Nelson CA 3rd. Effects of poverty on interacting biological systems underlying child development. Lancet Child Adolesc Health. 2017 Nov;1(3):225–39. doi: 10.1016/S2352-4642(17)30024-X 30169171

[9] Laquatra J, Maxwell LE, Pierce M. Indoor air pollutants: Limited-resource households and child care facilities. J Environ Health. 2005 Mar;67(7):39–43, 61. 15794462

[10] Evans GW. The physical context of child development. Curr Dir Psychol Sci. 2021 Feb 1;30(1):41–8.

[11] Dreger S, Schüle SA, Hilz LK, Bolte G. Social inequalities in environmental noise exposure: A review of evidence in the WHO European Region. Int J Environ Res Public Health [Internet]. 2019 Mar 20;16(6). Available from: doi: 10.3390/ijerph16061011 30897765 PMC6466273

[12] Elliott MR, Wang Y, Lowe RA, Kleindorfer PR. Environmental justice: Frequency and severity of US chemical industry accidents and the socioeconomic status of surrounding communities. J Epidemiol Community Health. 2004 Jan;58(1):24–30. doi: 10.1136/jech.58.1.24 14684723 PMC1757035

[13] McKenzie TL, Moody JS, Carlson JA, Lopez NV, Elder JP. Neighborhood income matters: Disparities in community recreation facilities, amenities, and programs. J Park Recreat Admi. 2013 Winter;31(4):12–22. doi: 10.1111/j.2047-6310.2013.00164.x 25006598 PMC4082954

[14] Coley RL, Leventhal T, Lynch AD, Kull M. Relations between housing characteristics and the well-being of low-income children and adolescents. Dev Psychol. 2013 Sep;49(9):1775–89. doi: 10.1037/a0031033 23244408 PMC3766502

[15] Leventhal T, Brooks-Gunn J. The neighborhoods they live in: The effects of neighborhood residence on child and adolescent outcomes. Psychol Bull. 2000 Mar;126(2):309–37. doi: 10.1037/0033-2909.126.2.309 10748645

[16] Hoff E, Laursen B. Socioeconomic status and parenting. In: Handbook of parenting. Routledge; 2019. p. 421–47.

[17] Conger RD, Martin MJ, Masarik AS. Dynamic associations among socioeconomic status (SES), parenting investments, and conscientiousness across time and generations. Dev Psychol. 2021 Feb;57(2):147–63. doi: 10.1037/dev0000463 33539124 PMC7869974

[18] Davis-Kean PE. The influence of parent education and family income on child achievement: The indirect role of parental expectations and the home environment. J Fam Psychol. 2005 Jun;19(2):294–304. doi: 10.1037/0893-3200.19.2.294 15982107

[19] Masarik AS, Conger RD. Stress and child development: a review of the Family Stress Model. Curr Opin Psychol. 2017 Feb;13:85–90. doi: 10.1016/j.copsyc.2016.05.008 28813301

[20] Davis-Kean PE, Tighe LA, Waters NE. The role of parent educational attainment in parenting and children’s development. Curr Dir Psychol Sci. 2021 Apr 1;30(2):186–92.

[21] Thomas MSC, Coecke S. Associations between socioeconomic status, cognition, and brain structure: Evaluating potential causal pathways through mechanistic models of development. Cogn Sci. 2023 Jan;47(1):e13217. doi: 10.1111/cogs.13217 36607218

[22] Rakesh D, Whittle S. Socioeconomic status and the developing brain—A systematic review of neuroimaging findings in youth. Neurosci Biobehav Rev. 2021 Nov;130:379–407. doi: 10.1016/j.neubiorev.2021.08.027 34474050

[23] Noble KG, Giebler MA. The neuroscience of socioeconomic inequality. Curr Opin Behav Sci. 2020 Dec;36:23–8. doi: 10.1016/j.cobeha.2020.05.007 32719820 PMC7384696

[24] Gur RE, Moore TM, Rosen AFG, Barzilay R, Roalf DR, Calkins ME, et al. Burden of environmental adversity associated with psychopathology, maturation, and brain behavior parameters in youths. JAMA Psychiatry. 2019 Sep 1;76(9):966–75. doi: 10.1001/jamapsychiatry.2019.0943 31141099 PMC6547104

[25] McDermott CL, Seidlitz J, Nadig A, Liu S, Clasen LS, Blumenthal JD, et al. Longitudinally mapping childhood socioeconomic status associations with cortical and subcortical morphology. J Neurosci. 2019 Feb 20;39(8):1365–73. doi: 10.1523/JNEUROSCI.1808-18.2018 30587541 PMC6381251

[26] Luby J, Belden A, Botteron K, Marrus N, Harms MP, Babb C, et al. The effects of poverty on childhood brain development: The mediating effect of caregiving and stressful life events. JAMA Pediatr. 2013 Dec;167(12):1135–42. doi: 10.1001/jamapediatrics.2013.3139 24165922 PMC4001721

[27] Noble Kimberly G., Houston Suzanne M., Brito Natalie H., Bartsch Hauke, Kan Eric, Kuperman Joshua M., et al. Family income, parental education and brain structure in children and adolescents. Nat Neurosci [Internet]. 2015; Available from: https://pubmed.ncbi.nlm.nih.gov/25821911 doi: 10.1038/nn.3983 25821911 PMC4414816

[28] Hanson JL, Chandra A, Wolfe BL, Pollak SD. Association between income and the hippocampus. PLoS One. 2011 May 4;6(5):e18712. doi: 10.1371/journal.pone.0018712 21573231 PMC3087752

[29] Moriguchi Y, Shinohara I. Socioeconomic disparity in prefrontal development during early childhood. Sci Rep. 2019 Feb 22;9(1):2585. doi: 10.1038/s41598-019-39255-6 30796284 PMC6385208

[30] Younger JW, Lee K-W, Demir-Lira OE, Booth JR. Brain lateralization of phonological awareness varies by maternal education. Dev Sci. 2019 Nov;22(6):e12807. doi: 10.1111/desc.12807 30735285

[31] Rakesh D, Zalesky A, Whittle S. Similar but distinct—Effects of different socioeconomic indicators on resting state functional connectivity: Findings from the Adolescent Brain Cognitive Development (ABCD) Study®. Dev Cogn Neurosci. 2021 Oct 1;51(101005):101005. 34419766 10.1016/j.dcn.2021.101005 PMC8379618

[32] Hanson JL, Albert WD, Skinner AT, Shen SH, Dodge KA, Lansford JE. Resting state coupling between the amygdala and ventromedial prefrontal cortex is related to household income in childhood and indexes future psychological vulnerability to stress. Dev Psychopathol. 2019 Aug;31(3):1053–66. doi: 10.1017/S0954579419000592 31084654

[33] Tomasi D, Volkow ND. Effects of family income on brain functional connectivity in US children: associations with cognition. Mol Psychiatry [Internet]. 2023 Aug 14; Available from: doi: 10.1038/s41380-023-02222-9 37580525

[34] Pietto ML, Kamienkowski JE, Lipina SJ. Electrophysiological approaches in the study of the influence of childhood poverty on cognition. In: Ibáñez A, Sedeño L, García AM, editors. Neuroscience and Social Science: The Missing Link. Cham: Springer International Publishing; 2017. p. 349–81.

[35] Olson L, Chen B, Fishman I. Neural correlates of socioeconomic status in early childhood: A systematic review of the literature. Child Neuropsychol. 2021 Apr 3;27(3):390–423. 33563106 10.1080/09297049.2021.1879766 PMC7969442

[36] Stevens C, Lauinger B, Neville H. Differences in the neural mechanisms of selective attention in children from different socioeconomic backgrounds: An event-related brain potential study. Dev Sci. 2009 Jul;12(4):634–46. doi: 10.1111/j.1467-7687.2009.00807.x 19635089 PMC2718768

[37] Giuliano RJ, Karns CM, Roos LE, Bell TA, Petersen S, Skowron EA, et al. Effects of early adversity on neural mechanisms of distractor suppression are mediated by sympathetic nervous system activity in preschool-aged children. Dev Psychol. 2018 Sep;54(9):1674–86. doi: 10.1037/dev0000499 30148395 PMC12614223

[38] Kishiyama MM, Boyce WT, Jimenez AM, Perry LM, Knight RT. Socioeconomic disparities affect prefrontal function in children. J Cogn Neurosci. 2009 Jun;21(6):1106–15. doi: 10.1162/jocn.2009.21101 18752394

[39] St. John AM, Tarullo AR. Neighbourhood chaos moderates the association of socioeconomic status and child executive functioning. Infant Child Dev. 2020 Jan;29(1). Available from: https://onlinelibrary.wiley.com/doi/abs/ doi: 10.1002/icd.2153

[40] Peters A, Zeytinoglu S, Leerkes EM, Isbell E. Component-specific developmental trajectories of ERP indices of cognitive control in early childhood. Dev Cogn Neurosci. 2023 Dec 1;64:101319. doi: 10.1016/j.dcn.2023.101319 37907010 PMC10632416

[41] Dufford AJ, Evans GW, Dmitrieva J, Swain JE, Liberzon I, Kim P. Prospective associations, longitudinal patterns of childhood socioeconomic status, and white matter organization in adulthood. Hum Brain Mapp. 2020 Sep;41(13):3580–93. doi: 10.1002/hbm.25031 32529772 PMC7416042

[42] Javanbakht A, King AP, Evans GW, Swain JE, Angstadt M, Phan KL, et al. Childhood poverty predicts adult amygdala and frontal activity and connectivity in response to emotional faces. Front Behav Neurosci. 2015 Jun 12;9:154. doi: 10.3389/fnbeh.2015.00154 26124712 PMC4464202

[43] Dufford AJ, Evans GW, Liberzon I, Swain JE, Kim P. Childhood socioeconomic status is prospectively associated with surface morphometry in adulthood. Dev Psychobiol. 2021 Jul;63(5):1589–96. doi: 10.1002/dev.22096 33432574 PMC13102113

[44] Gianaros PJ, Horenstein JA, Hariri AR, Sheu LK, Manuck SB, Matthews KA, et al. Potential neural embedding of parental social standing. Soc Cogn Affect Neurosci. 2008 Jun;3(2):91–6. doi: 10.1093/scan/nsn003 18594696 PMC2311502

[45] Chen C, Wang Z, Cao X, Zhu J. Exploring the association between early exposure to material hardship and psychopathology through indirect effects of fronto-limbic functional connectivity during fear learning. Cereb Cortex. 2023 Sep 8;33(20):10702–10. doi: 10.1093/cercor/bhad320 37689831

[46] Hoebel J, Lampert T. Subjective social status and health: Multidisciplinary explanations and methodological challenges. J Health Psychol. 2020 Feb;25(2):173–85. doi: 10.1177/1359105318800804 30230391

[47] Adler NE, Epel ES, Castellazzo G, Ickovics JR. Relationship of subjective and objective social status with psychological and physiological functioning: Preliminary data in healthy white women. Health Psychol. 2000 Nov;19(6):586–92. doi: 10.1037//0278-6133.19.6.586 11129362

[48] Tan JJX, Kraus MW, Carpenter NC, Adler NE. The association between objective and subjective socioeconomic status and subjective well-being: A meta-analytic review. Psychol Bull. 2020 Nov;146(11):970–1020. doi: 10.1037/bul0000258 33090862

[49] Galvan MJ, Payne BK, Hannay J, Georgeson AR, Muscatell KA. What does the MacArthur Scale of Subjective Social Status measure? Separating economic circumstances and social status to predict health. Ann Behav Med. 2023 Oct 16;57(11):929–41. doi: 10.1093/abm/kaad054 37742041

[50] Goodman E, Maxwell S, Malspeis S, Adler N. Developmental trajectories of subjective social status. Pediatrics. 2015 Sep;136(3):e633–40. doi: 10.1542/peds.2015-1300 26324868 PMC4552092

[51] Destin M, Richman S, Varner F, Mandara J. “Feeling” hierarchy: The pathway from subjective social status to achievement. J Adolesc. 2012 Dec;35(6):1571–9. doi: 10.1016/j.adolescence.2012.06.006 22796063 PMC3490056

[52] Gianaros PJ, Horenstein JA, Cohen S, Matthews KA, Brown SM, Flory JD, et al. Perigenual anterior cingulate morphology covaries with perceived social standing. Soc Cogn Affect Neurosci. 2007 Sep;2(3):161–73. doi: 10.1093/scan/nsm013 18418472 PMC2312334

[53] Muscatell KA, Dedovic K, Slavich GM, Jarcho MR, Breen EC, Bower JE, et al. Neural mechanisms linking social status and inflammatory responses to social stress. Soc Cogn Affect Neurosci. 2016 Jun;11(6):915–22. doi: 10.1093/scan/nsw025 26979965 PMC4884319

[54] Ly M, Haynes MR, Barter JW, Weinberger DR, Zink CF. Subjective socioeconomic status predicts human ventral striatal responses to social status information. Curr Biol. 2011 May 10;21(9):794–7. doi: 10.1016/j.cub.2011.03.050 21530264

[55] Kappenman ES, Farrens JL, Zhang W, Stewart AX, Luck SJ. ERP CORE: An open resource for human event-related potential research. Neuroimage. 2021 Jan 15;225:117465. doi: 10.1016/j.neuroimage.2020.117465 33099010 PMC7909723

[56] Näätänen R, Kujala T, Light G. Mismatch Negativity: A Window to the Brain. Oxford University Press; 2019. 288 p.

[57] Sussman ES, Chen S, Sussman-Fort J, Dinces E. The five myths of MMN: Redefining how to use MMN in basic and clinical research. Brain Topogr. 2014 Jul;27(4):553–64. doi: 10.1007/s10548-013-0326-6 24158725 PMC4000291

[58] Näätänen R, Kreegipuu K. The mismatch negativity (MMN). In: Oxford Handbook of Event-Related Potentials. Oxford University Press; 2013. p. 143–57.

[59] Putkinen V, Tervaniemi M, Saarikivi K, de Vent N, Huotilainen M. Investigating the effects of musical training on functional brain development with a novel Melodic MMN paradigm. Neurobiol Learn Mem. 2014 Apr;110:8–15. doi: 10.1016/j.nlm.2014.01.007 24462719

[60] Tamminen H, Peltola MS, Kujala T, Näätänen R. Phonetic training and non-native speech perception—New memory traces evolve in just three days as indexed by the mismatch negativity (MMN) and behavioural measures. Int J Psychophysiol. 2015 Jul;97(1):23–9. doi: 10.1016/j.ijpsycho.2015.04.020 25956191

[61] Polich J. Neuropsychology of P300. The Oxford handbook of event-related potential components. 2012;159–88.

[62] Polich J. Updating P300: An integrative theory of P3a and P3b. Clin Neurophysiol. 2007 Oct;118(10):2128–48. doi: 10.1016/j.clinph.2007.04.019 17573239 PMC2715154

[63] St John AM, Finch K, Tarullo AR. Socioeconomic status and neural processing of a go/no-go task in preschoolers: An assessment of the P3b. Dev Cogn Neurosci. 2019;100677. doi: 10.1016/j.dcn.2019.100677 31255904 PMC6969333

[64] United States Census Bureau. Survey of Income and Program Participation (SIPP) [Internet]. 2022. Available from: https://www.census.gov/programs-surveys/sipp.html

[65] actiCHamp Plus (64 channels) [Apparatus]. (2019). Gilching, Germany: Brain Products GmbH.

[66] Delorme A, Makeig S. EEGLAB: An open source toolbox for analysis of single-trial EEG dynamics including independent component analysis. J Neurosci Methods. 2004 Mar 15;134(1):9–21. doi: 10.1016/j.jneumeth.2003.10.009 15102499

[67] Lopez-Calderon J, Luck SJ. ERPLAB: An open-source toolbox for the analysis of event-related potentials. Front Hum Neurosci. 2014 Apr 14;8:213. doi: 10.3389/fnhum.2014.00213 24782741 PMC3995046

[68] Pion-Tonachini L, Kreutz-Delgado K, Makeig S. ICLabel: An automated electroencephalographic independent component classifier, dataset, and website. Neuroimage. 2019 Sep;198:181–97. doi: 10.1016/j.neuroimage.2019.05.026 31103785 PMC6592775

[69] Isbell E, Grammer JK. Event‐related potentials data quality in young children: Standardized measurement error of ERN and Pe. Dev Psychobiol [Internet]. 2022 Feb;64(4). Available from: https://onlinelibrary.wiley.com/doi/10.1002/dev.22245 35452543 10.1002/dev.22245

[70] Luck SJ, Stewart AX, Simmons AM, Rhemtulla M. Standardized measurement error: A universal metric of data quality for averaged event-related potentials. Psychophysiology. 2021 Mar 29;e13793. doi: 10.1111/psyp.13793 33782996 PMC8169536

[71] Zhang W, Kappenman ES. Maximizing signal-to-noise ratio and statistical power in ERP measurement: Single sites versus multi-site average clusters. Psychophysiology. 2023 Nov 16;e14440. doi: 10.1111/psyp.14440 37973199

[72] Luck SJ. An Introduction to the Event-Related Potential Technique. MIT Press; 2014. 416 p.

[73] Stanislaw H, Todorov N. Calculation of signal detection theory measures. Behav Res Methods Instrum Comput. 1999 Feb;31(1):137–49. doi: 10.3758/bf03207704 10495845

[74] Tabachnick BG, Fidell LS. Using multivariate statistics. Vol. 5. Pearson Boston, MA; 2007.

[75] Hampton Wray A, Stevens C, Pakulak E, Isbell E, Bell T, Neville H. Development of selective attention in preschool-age children from lower socioeconomic status backgrounds. Dev Cogn Neurosci. 2017 Aug 1;26:101–11. doi: 10.1016/j.dcn.2017.06.006 28735165 PMC5703215

[76] Rosen ML, Hagen MP, Lurie LA, Miles ZE, Sheridan MA, Meltzoff AN, et al. Cognitive stimulation as a mechanism linking socioeconomic status with executive function: A longitudinal investigation. Child Dev. 2020 Jul;91(4):e762–79. doi: 10.1111/cdev.13315 31591711 PMC7138720

[77] Tighe LA, Davis-Kean PE. The influence of college education on parents and children in low-income families. mpq. 2021;67(3):293–328.

[78] Zocher S, Schilling S, Grzyb AN, Adusumilli VS, Bogado Lopes J, Günther S, et al. Early-life environmental enrichment generates persistent individualized behavior in mice. Sci Adv. 2020 Aug;6(35):eabb1478. doi: 10.1126/sciadv.abb1478 32923634 PMC7449688

[79] Ellwood-Lowe ME, Whitfield-Gabrieli S, Bunge SA. Brain network coupling associated with cognitive performance varies as a function of a child’s environment in the ABCD study. Nat Commun. 2021 Dec 10;12(1):7183. doi: 10.1038/s41467-021-27336-y 34893612 PMC8664837

[80] Leonard JA, Romeo RR, Park AT, Takada ME, Robinson ST, Grotzinger H, et al. Associations between cortical thickness and reasoning differ by socioeconomic status in development. Dev Cogn Neurosci. 2019 Apr;36:100641. doi: 10.1016/j.dcn.2019.100641 30951970 PMC6969225

[81] Romeo RR, Perrachione TK, Olson HA, Halverson KK, Gabrieli JDE, Christodoulou JA. Socioeconomic dissociations in the neural and cognitive bases of reading disorders. Dev Cogn Neurosci. 2022 Dec;58:101175. doi: 10.1016/j.dcn.2022.101175 36401889 PMC9674867

[82] Dunkel Schetter C, Schafer P, Lanzi RG, Clark-Kauffman E, Raju TNK, Hillemeier MM, et al. Shedding light on the mechanisms underlying health disparities through community participatory methods: The stress pathway. Perspect Psychol Sci. 2013 Nov;8(6):613–33. doi: 10.1177/1745691613506016 26173227 PMC4505627

[83] Garrett-Peters PT, Mokrova I, Vernon-Feagans L, Willoughby M, Pan Y, Family Life Project Key Investigators. The role of household chaos in understanding relations between early poverty and children’s academic achievement. Early Child Res Q. 2016;37:16–25. 27330247 10.1016/j.ecresq.2016.02.004 PMC4909052

[84] Amir D, Valeggia C, Srinivasan M, Sugiyama LS, Dunham Y. Measuring subjective social status in children of diverse societies. PLoS One. 2019 Dec 20;14(12):e0226550. doi: 10.1371/journal.pone.0226550 31860691 PMC6924674

[85] Peretz-Lange R, Harvey T, Blake PR. From “haves” to “have nots”: Developmental declines in subjective social status reflect children’s growing consideration of what they do not have. Cognition. 2022 Jun;223:105027. doi: 10.1016/j.cognition.2022.105027 35124455

