# Supplementary material for: Childhood family socioeconomic status is linked to adult brain electrophysiology
Source: PLoS One. 2024 Aug 20;19(8):e0307406. doi: 10.1371/journal.pone.0307406 (PMC11335154; doi:10.1371/journal.pone.0307406)
Supplement: S1 Text — (DOCX) [file pone.0307406.s001.docx]

**S1 Text. Supplementary Information**

**The tables and figures appear in the order they appear in the main manuscript.**

**Passive auditory oddball task**

Initially, we planned to use the original ERP CORE task [1]. However, when we started collecting data using the original task (*n* = 25), approximately 3-5 minutes after the initiation of the task, several participants displayed posterior slow wave onset, which is considered a neuroelectrophysiological characteristic of task disengagement and transitioning to sleep [2]. We also recognized some participants were visibly getting sleepy, as observed via the monitoring camera we have in our booth. The timing of this observation overlapped with the preparation of a child-friendly version of the task in our lab to be used in cross-sectional studies with a wide age range. In this child-friendly version, stimuli and the interstimulus interval were kept identical to ERP CORE. However, there were two differences between the ERP CORE task and the child-friendly auditory oddball task we used in this study. First, we replaced the “Sand Art” video used in ERP CORE with a more engaging animation, which we thought could be used not only with adults but also with children (i.e., “Pingu” the penguin). Second, we reduced the duration of the task from 10 minutes to approximately 3.5 minutes. Previous research demonstrated that MMN can be elicited with as few as 25 rare stimuli and 165 total stimuli in children and adults [3]. We aimed to adapt the original ERP CORE task to be as short as possible with the same frequent:rare stimuli ratio as the original task, while maximizing the amount of deviant stimuli to elicit MMN. With the same 80:20 frequent:rare stimuli ratio, 350 total stimuli allowed for 70 deviant stimuli to be administered. MMN can typically be influenced by changes in stimuli properties and changes to the interstimulus interval [4,5]. Thus, our stimuli and the interstimulus interval were kept identical to Kappenman et al. (2021) to elicit a robust MMN similar to that observed in the original ERP CORE study. The modifications to the task allowed us to have a more engaging and shorter task for use with a broad age range from young children to adults, while allowing us to elicit a robust MMN response. The MMN elicited during the original ERP CORE passive auditory oddball task is depicted in Fig D grand-average ERP plot for the ERP CORE sample (1) and in Fig E grand-average ERP plot for a pilot sample collected in our lab. The MMN elicited during our modified passive auditory oddball task is depicted in Fig F grand-average ERP plot.

Table A. Descriptives of sociodemographic characteristics for participants with one or two usable ERP tasks.

|  | n | | | Mean | SD |
| --- | --- | --- | --- | --- | --- |
| Age  One task  Both tasks | | 24  62 | 21.20  22.10 | | 1.95  2.96 |
| Participant education (years)  One task  Both tasks | | 24  62 | 13.17  13.65 | | 1.63  1.78 |
| Childhood parent education (years)  One task  Both tasks | | 24  62 | 13.13  12.29 | | 4.73  4.07 |
| Childhood subjective family SES  One task  Both tasks | | 24  62 | 5.21  4.77 | | 2.00  2.11 |

Note. One task = has usable ERP data from either the passive auditory or active visual oddball task; Both tasks = have usable ERP data from both tasks.

Table B. Independent samples t-test comparing the sociodemographic characteristics of participants with one or two usable ERP tasks.

|  | t | *p* | Confidence Interval (95%) | | Cohen’s d |
| --- | --- | --- | --- | --- | --- |
|  |  |  | Lower | Upper |  |
| Age | -1.37 | .17 | -2.20 | .40 | -.33 |
| Participant education | 1.14 | .26 | -1.31 | .36 | -.27 |
| Childhood parent education | .81 | .42 | -1.20 | 2.87 | .20 |
| Childhood subjective family SES | .87 | .39 | -.56 | 1.43 | .21 |

Table C. Descriptives for artifact-free ERP trials, ERP mean amplitudes, and aSME values for frequent and rare trials in the auditory oddball (MMN) and visual oddball (P3b) tasks.

| Variables | Mean | SD | Min | Max |
| --- | --- | --- | --- | --- |
| MMN ERP trials  Frequent  Rare | 191.14  68.56 | 5.19  2.18 | 170  60 | 196  70 |
| MMN mean amplitude (µV)  Frequent  Rare | 0.70  -1.45 | 1.32  1.69 | -2.50  -5.23 | 3.47  2.11 |
| MMN aSME  Frequent  Rare | 0.61  1.02 | 0.09  0.18 | 0.46  0.67 | 0.87  1.48 |
| P3b ERP trials  Frequent  Rare | 149.47  33.13 | 16.46  4.47 | 68  17 | 160  40 |
| P3b mean amplitude (µV)  Frequent  Rare | 6.34  12.30 | 2.96  5.11 | 0.45  1.84 | 17.61  33.53 |
| P3b aSME  Frequent  Rare | 0.87  1.75 | 0.20  0.48 | 0.58  1.06 | 1.58  2.95 |

Fig A. Frequencies of participant educational attainment in years for the analytic sample (N=86).

Fig B. Frequencies of childhood parent educational attainment in years for the analytic sample (N=86).

Fig C. Frequencies of childhood subjective family SES for the analytical sample (N=86). Higher values denote higher subjective ratings of family SES.


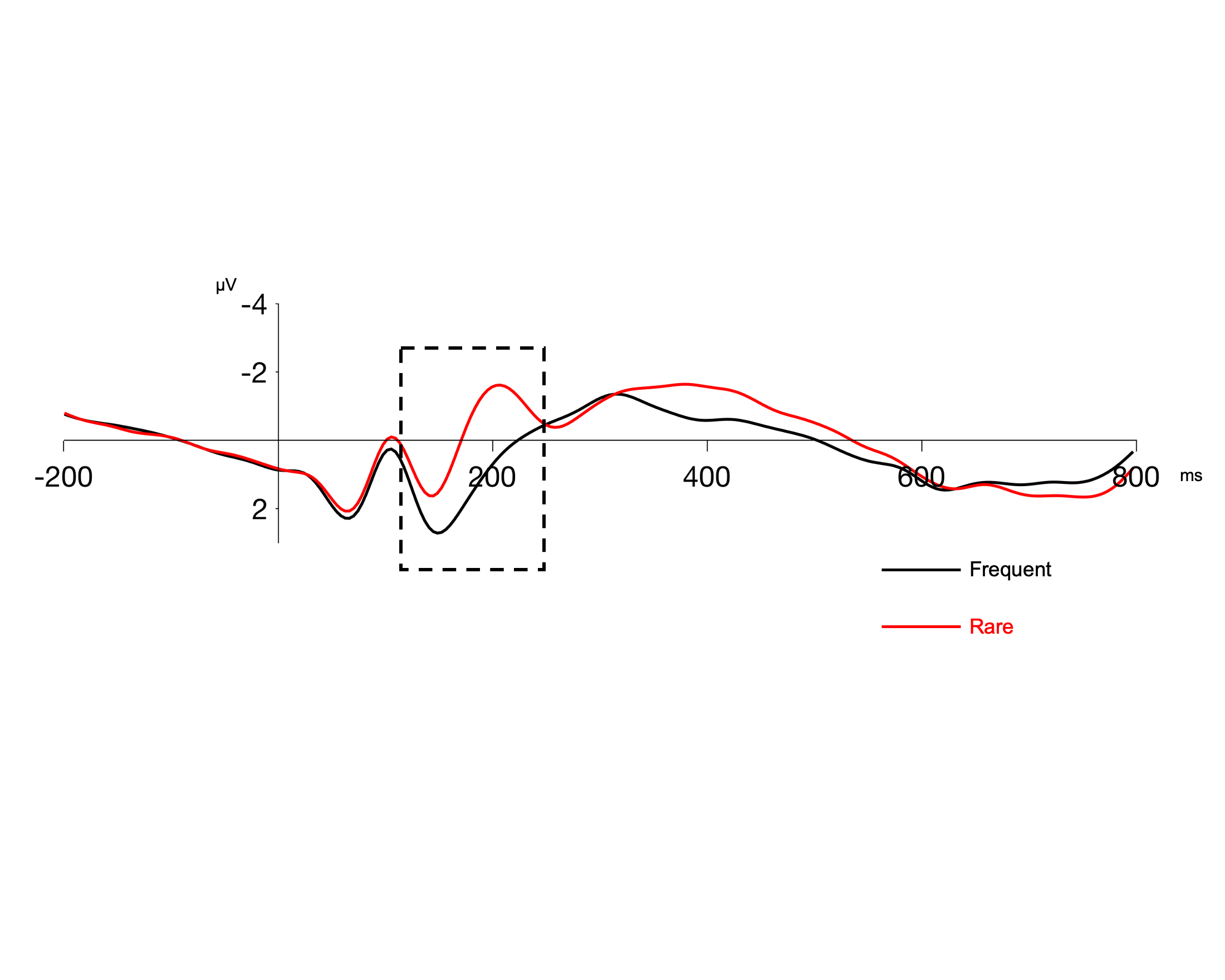


Fig D. Cluster grand-average ERP plot for MMN for the data reported in ERP CORE (Kappenman et al., 2021), with the original passive auditory oddball task (*n* = 39). A frontocentral electrode cluster was created for direct comparison with the grand-average plots included in the present study (F3, Fz, F4, C3, C4). Due to differences in electrode configurations across labs, FC1 and FC2 could not be included in the frontocentral cluster. By convention, negative is plotted upward. The MMN measurement window is shown in the dotted rectangle (125-225 ms post-stimulus onset). MMN was measured as the difference between Rare (red waveform) and Frequent (black waveform) conditions.


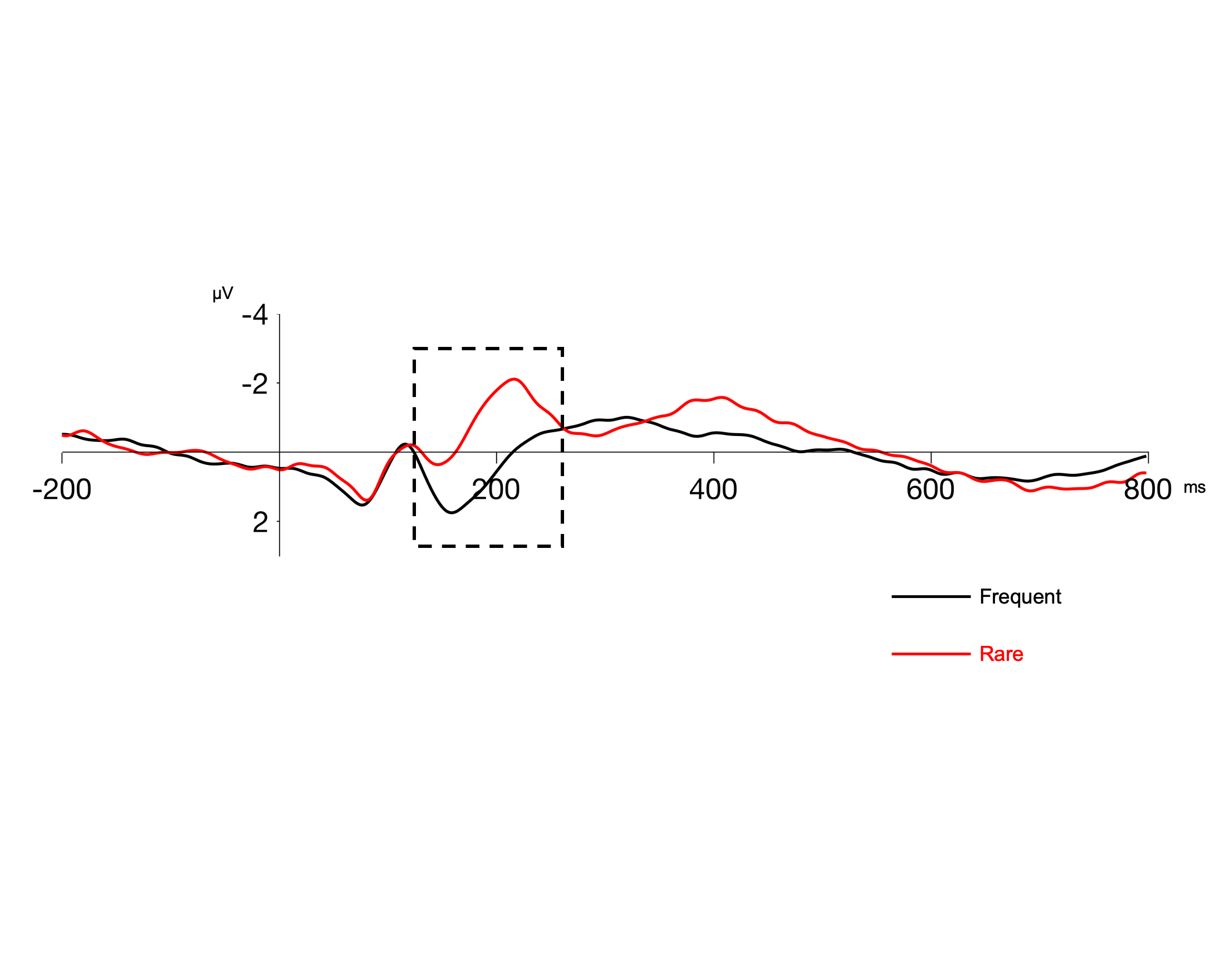


Fig E. Cluster grand-average ERP plot for MMN for the original passive auditory oddball task collected from a pilot sample in our lab (*n* = 25). ERPs were measured over a frontocentral electrode cluster (F3, Fz, F4, FC1, FC2, C3, C4). By convention, negative is plotted upward. The MMN measurement window is shown in the dotted rectangle (125-225 ms post-stimulus onset). MMN was measured as the difference between Rare (red waveform) and Frequent (black waveform) conditions.


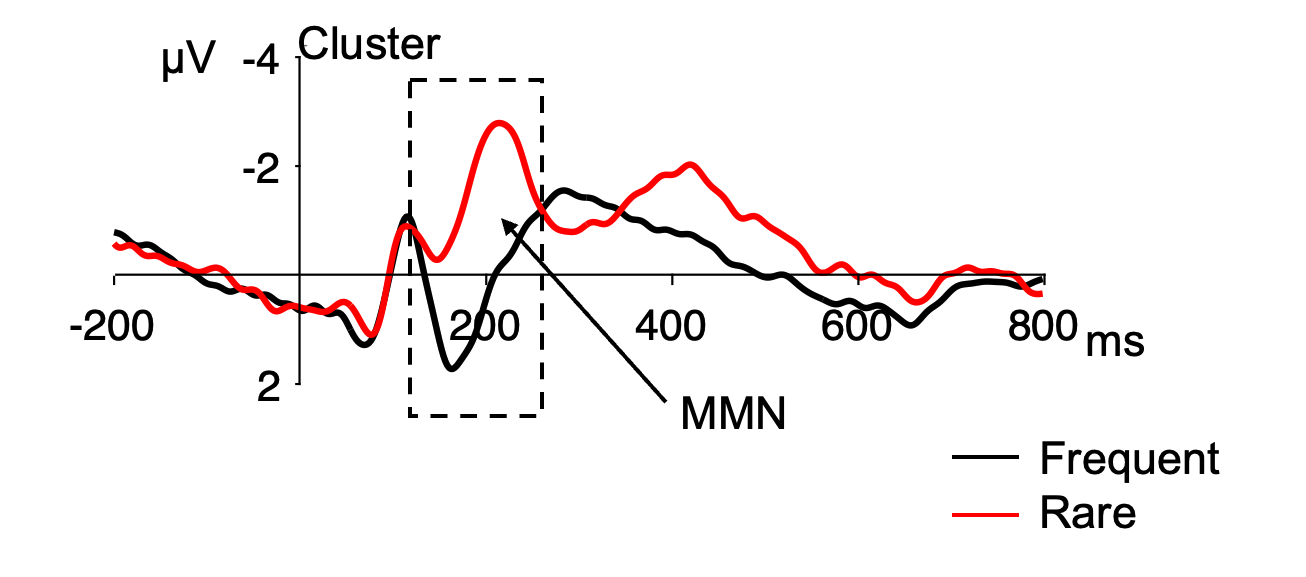


Fig F. Cluster grand-average ERP plot for MMN (*n* = 73). By convention, negative is plotted upward. The MMN measurement window is shown in the dotted rectangle (125-225 ms post-stimulus onset). MMN was measured as the difference between Rare (red waveform) and Frequent (black waveform) conditions.


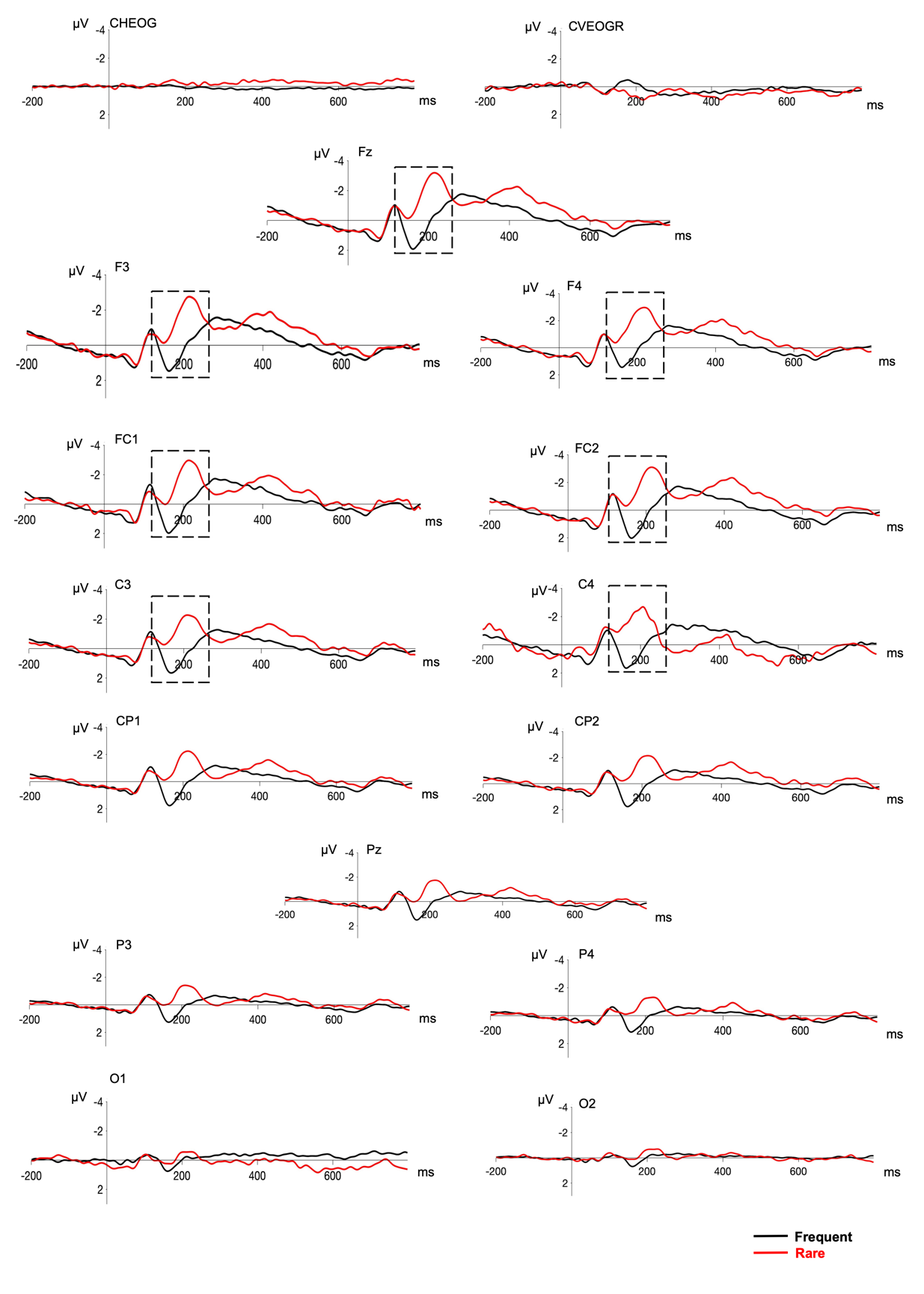


Fig G. Grand-average ERP plot for MMN in the passive auditory oddball task used in the study (n = 73), depicted over representative channels across the scalp. CHEOG= Artifact-corrected horizontal bipolar electrooculogram channel; CVEOGR= Artifact-corrected right vertical bipolar electrooculogram channel. By convention, negative is plotted upward. The MMN measurement window is shown with a dotted rectangle (125-225 ms post-stimulus onset) over electrodes that were included in the frontocentral cluster. MMN was measured as the difference between Rare (red waveform) and Frequent (black waveform) conditions.


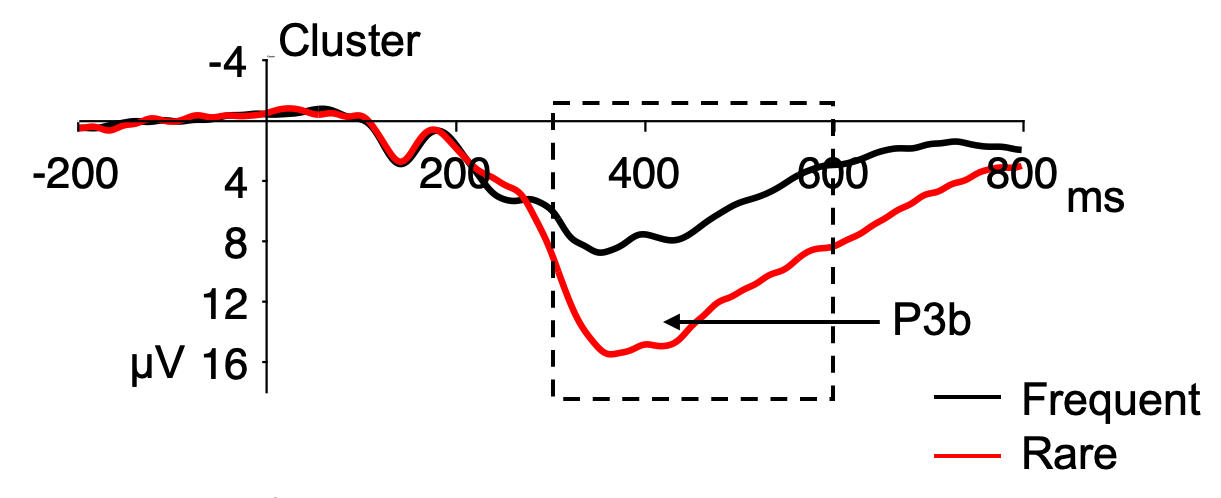


Fig H. Cluster grand-average ERP plot for P3b for frequent (black waveform) and rare (red waveform) conditions in the visual oddball task (n = 75). By convention, negative is plotted upward. The P3b measurement window is shown with a dotted square (300-600 ms post-stimulus onset).

**
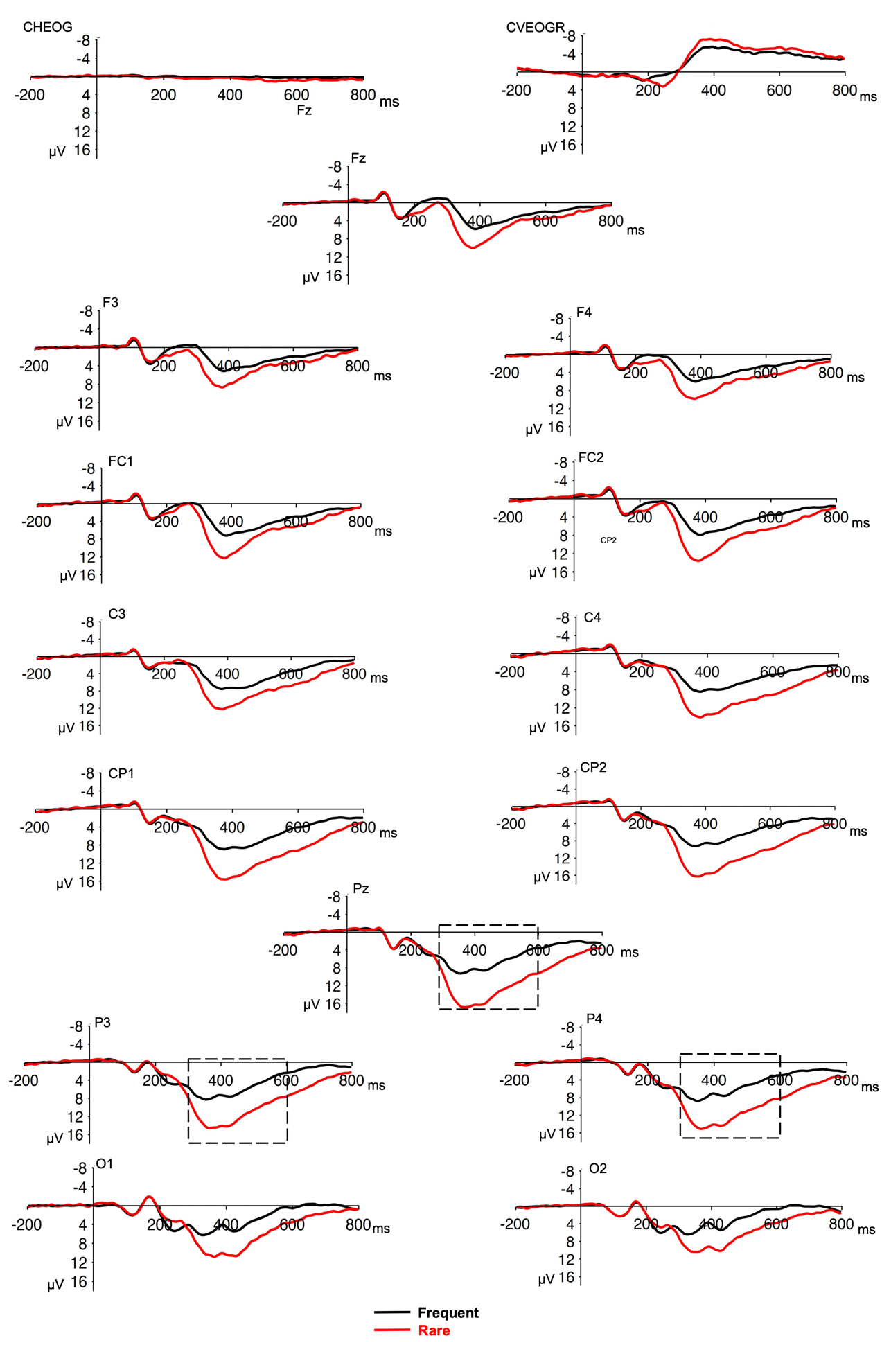
**

Fig I. Grand-average ERP plots for P3b for Frequent (black waveform) and Rare (red waveform) conditions in the visual oddball task (n = 75) over representative channels across the scalp. CHEOG= Artifact-corrected horizontal bipolar electrooculogram channel; CVEOGR= Artifact-corrected right vertical bipolar electrooculogram channel. By convention, negative is plotted upward. The P3b measurement window is shown with a dotted rectangle (300-600 ms post-stimulus onset) over electrodes that were included in the parietal cluster.

**Appendix A. Childhood parent education questionnaire.**

Please answer the following questions for **your parents/legal guardians (e.g., mother, father, grandparent, stepparent, etc.)** when you were **10 years-old.**

**Adult 1**

Relationship to you (mother, father, grandparent, stepparent, etc.): ____________

What was the highest level of education completed by this person when you were 10 years-old?

- Less than 1^st^ grade
- 1^st^, 2^nd^, 3^rd^, or 4^th^ grade
- 5^th^ or 6^th^ grade
- 7^th^ or 8^th^ grade
- 9^th^ grade
- 10^th^ grade
- 11^th^ grade
- 12^th^ grade, no diploma
- High School Graduate (diploma or GED or equivalent)
- Some college credit, but less than 1 year
- 1 or more years of college, no degree
- Associate degree (2-year college)
- Bachelor’s degree (for example: BA, BS)
- Master’s degree (for example: MA, MS, MBA, MFA, MSW)
- Professional School Degree (for example: MD, DDS, JD)
- Doctorate Degree (for example: PhD, Ed.D.)

Was this person living in your household when you were 10 years-old?

- Yes
- No

**Coding for parent education levels**

The categorical descriptions of parental educational attainment were converted into years of education to evaluate childhood parent education as a continuous variable. The coding scheme is outlined below:

- Less than 1^st^ grade = 0 years
- 1^st^, 2^nd^, 3^rd^, or 4^th^ grade = 4 years
- 5^th^ or 6^th^ grade = 6 years
- 7^th^ or 8^th^ grade = 8 years
- 9^th^ grade = 9 years
- 10^th^ grade = 10 years
- 11^th^ grade = 11 years
- 12^th^ grade, no diploma = 11 years
- High School Graduate (Diploma or GED or equivalent) = 12 years
- Some college credit, but less than 1 year = 13 years
- 1 or more years of college, no degree = 13 years
- Associate degree (2-year college) = 14 years
- Bachelor’s degree (for example: BA, BS) = 16 years
- Master’s degree (for example: MA, MS, MBA, MFA, MSW) = 18 years
- Professional School Degree (for example: MD, DDS, JD) = 19
- Doctorate Degree (for example: PhD, Ed.D.) = 20

**Childhood family subjective socioeconomic status.**

**Instructions:** Imagine that this ladder pictures how **American society is set up**.

At the top of the ladder are the people who are the best off — those who have the most money, the highest amount of schooling, and the jobs that bring the most respect. At the bottom are people who are the worst off — those who have the least money, little or no education, no job, or jobs that no one wants or respects.

Now think about your family **when you were 10 years-old.**

Please tell us where you think your family would be on this ladder. Mark the rung that best represents where your family would be on this ladder.


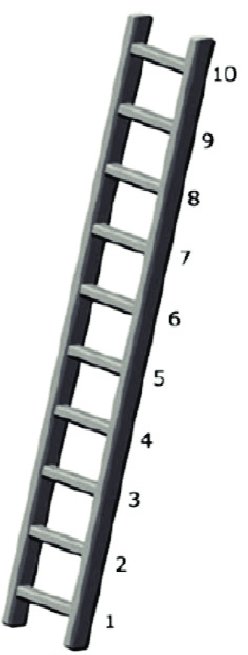


Note: Childhood subjective social status questionnaire was adapted from Adler et al. (2000).

**Supplementary Information References**

1. Kappenman ES, Farrens JL, Zhang W, Stewart AX, Luck SJ. ERP CORE: An open resource for human event-related potential research. Neuroimage. 2021 Jan 15;225:117465.

2. Andrillon T, Burns A, Mackay T, Windt J, Tsuchiya N. Predicting lapses of attention with sleep-like slow waves. Nat Commun. 2021 Jun 29;12(1):3657.

3. Kraus N, McGee T, Sharma A, Carrell T, Nicol T. Mismatch negativity event-related potential elicited by speech stimuli. Ear Hear. 1992 Jun;13(3):158–64.

4. Näätänen R, Pakarinen S, Rinne T, Takegata R. The mismatch negativity (MMN): Towards the optimal paradigm. Clin Neurophysiol. 2004 Jan;115(1):140–4.

5. Näätänen R, Kujala T, Light G. Mismatch Negativity: A Window to the Brain. Oxford University Press; 2019. 288 p.
